# TAUS: Target‐Age Unified Survival. Survival Analysis Without Assuming Proportional Hazards or Parameterising the Survival Function

**DOI:** 10.1002/ece3.74034

**Published:** 2026-09-07

**Authors:** Iván Casas Gomez‐Uribarri, Simon A. Babayan, Fredros Okumu, Francesco Baldini, Mauro Pazmiño Betancourth

**Affiliations:** ^1^ School of Biodiversity, One Health & Veterinary Medicine University of Glasgow Glasgow UK; ^2^ Ifakara Health Institute Ifakara Morogoro Region Tanzania

**Keywords:** conditional probability, Cox proportional hazards, demography, Kaplan–Meier, parametric survival models, R package, survival analysis, TAUS

## Abstract

Standard survival analyses often assume proportional hazards (PH) or impose parametric survival functions that poorly represent real populations. Ecological applications are further limited by missing age data. Since standard survival methods estimate probabilities of survival at birth, this introduces an important survivorship bias. To circumvent these modelling constraints, we developed a method that combines the Kaplan–Meier estimator with conditional probability theory to compute age‐specific probabilities of survival up to some target age of choice τ. Marginalising this probability over the age distribution of the population yields $O_{\tau }$, the probability that a randomly sampled individual of unknown age will outlive the target age τ. Notably, τ is set for each group independently, which allows accounting for differences in pace of life across populations. We tested its application using a simulation study and two real‐world datasets, and compared its performance against that of Cox PH and parametric survival models. The PH assumption was violated in the three examples, rendering the Cox PH models inappropriate. Parametric models offered a better alternative, but the best parametric fit missed at least some key survival patterns in all examples. The TAUS method provided a valid description of survival patterns in all cases. Its output also allowed finer analysis of survival differences between populations. The TAUS method is also available as an R package (https://github.com/casasgomezuribarri/TAUS). This new method, free of the PH and parametric assumptions, allows the comparison of survival probabilities between populations with different age structures and rates of pace of life. This makes it suitable for a wide range of ecological applications, including in population viability analysis, epidemiology, or life‐history theory.

## Graphical abstract

Estimation of 𝑂_𝜏_ for the arbitrary case of 𝜏 = 15 (green dashed line). It shows the conditional survival (𝑇 > 𝜏 ∣ 𝑇 > 𝑡) (black line, 95% CI in grey), the age distribution 𝐴(𝑡) (blue histogram), the *t*‐wise evaluation of 𝑃(𝑇 > 𝜏 ∣ 𝑇 > 𝑡) * 𝐴(𝑡) (red line), and the value of 𝑂_𝜏_ = ∑_𝑡_ 𝑃(𝑇 > 𝜏 ∣ 𝑇 > 𝑡) ⋅ 𝐴(𝑡) (red area).

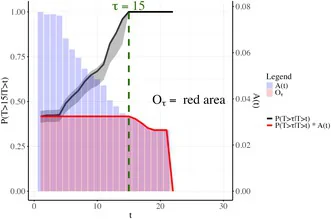

## 1 Introduction

Survival analysis is the statistical study of time‐to‐event data, where the outcome variable is the time between two observable events. In practice, the first event is usually the start of the study or process, and the outcome is the time until the event of interest occurs. It is the convention to refer to this event of interest as *death*, although its nature is not restricted to biological death. The terminology is coherent with this convention: *birth* refers to the start of the process under study, *age* is the time since birth and *lifespan* is the typical age at death.

Understanding how survival patterns change across populations, time and environmental conditions is central to many questions in ecology and evolutionary biology, with direct applications in conservation, epidemiology, or life‐history theory (Nur et al. 2004; Scherm and Ojiambo 2007; Selvin 2010; Heppell 1998). However, the most common methods for survival analysis—Cox proportional hazards (PH) and parametric survival models—are often inadequate for use in ecological contexts.

Cox PH models investigate the effect of one or more explanatory variables on survival by estimating the ratio of the hazards of different groups, where hazard is defined as the age‐specific immediate risk of death, given survival to that age (Cox 1972). A central assumption of the model is that this ratio is constant over time, but this is often violated in practice (Kuitunen et al. 2021; Dunkler et al. 2018; Kleinbaum and Klein 2012; Ishwaran et al. 2008; Gensheimer and Narasimhan 2019; Tang et al. 2022). Methods to relax this limitation include stratifying the data by the variable that violates the assumption (Kleinbaum and Klein 2012), partitioning the timeline into smaller intervals for which the hazards ratio is assumed to be constant (Kuitunen et al. 2021), or applying weights to different timepoints to estimate the *average* hazard ratio (Dunkler et al. 2018). Despite these extensions, Cox PH models remain limited to relative descriptions of survivorship—providing no absolute estimates of individual hazards—and inherently unable to describe how covariate effects change over time. More specifically: stratifying by the variable that violates the assumption removes the ability to quantify its effect; timeline partitioning makes the results dependent on the specific segments chosen, and the relaxation of the PH assumption may be explained by its lower detectability due to the decrease in sample size and follow‐up period of each time segment; applying time weights to estimate an average hazard ratio over time naturally prevents the investigation of time‐varying effects; and including arbitrary functions of time in the set of predictor variables introduces circularity issues, since the response variable of interest is also time. Notably, absolute survival estimates and time‐varying effects are often of interest in ecological applications of survival analysis. Indeed, Cox himself noted the difficulty of giving a clear physical or biological interpretation of the model's output, and later expressed a preference for parametric approaches when analysing censored survival data (Cox 1997; Cox and Reid 1994).

Parametric survival models, the second most common approach to survival analysis, assume that survival times follow some known probability distribution and then estimate its parameters as a function of the explanatory variables (Crowther and Lambert 2014). Unlike the Cox PH model, they can produce absolute survival estimates and detect effects that are time‐dependent (Crowther and Lambert 2013). However, they rely on the good fit of the chosen distribution or spline configuration for the baseline hazard function and consequently inherit its structural constraints. These include restrictions on modality, hazard shape, parameterisation, and on distributional properties such as symmetry or tail behaviour. It has been shown that, in this context, model misspecification can cause bias of clinically relevant magnitude in medical applications of survival analysis (Bellera et al. 2010; Nardi and Schemper 2003; Gasparini et al. 2018). Parametric survival models are also limited in their ability to compare survival dynamics across populations that follow different distributions, even when their independent fits are adequate.

More recent survival methods exist that aim to relax these assumptions (e.g., Qi et al. 2024; Gensheimer and Narasimhan 2019; Yu et al. 2011; Ishwaran et al. 2008; Tang et al. 2022; Rindt et al. 2022). However, most were developed with clinical applications of survival analysis in mind. In these cases, there is often a wealth of data available about each patient (medical history, lifestyle, specific biomarkers), and new models have focused on using these data to personalise prognosis, which is an improvement from population‐based estimates in health care applications of survival. As a result, many new models use machine learning (Ishwaran et al. 2008; Rindt et al. 2022; Tang et al. 2022; Gensheimer and Narasimhan 2019) to disentangle complex patterns. These algorithmic choices might not be the most appropriate in ecological applications of survival analysis, where data on each individual are scarce (i.e., risk of overfitting) and mechanistic understanding is of high value. Additionally, in clinical settings, survival assessment always happens at the time of diagnosis (i.e., *t* = 0), whereas a common task in ecology is to estimate survival of sampled individuals (i.e., t≠0, often not known). Since survival functions usually estimate survival probabilities at birth, this introduces a strong survivorship bias in ecological applications of survival analysis. In summary, clinical and ecological applications of survival analysis represent substantially different problems in terms of data structure, applications, challenges and aims. As a result, models developed with one use‐case in mind might not be readily applicable to the other.

Here, we present a novel approach to survival analysis that does not parameterise the hazard function or assume proportional hazards. Our method produces absolute survival estimates, which come in the form of the predicted probability of outliving some age τ. More specifically, it quantifies the probability that a *randomly sampled individual from a population of known (or estimated) age distribution, but whose current age is unknown* will live to be at least age τ. This metric is particularly useful in ecology, or more broadly in contexts where:

- Estimating the age structure of a population may be more feasible and cost‐effective than age‐grading every individual, which is often the case when working with wild populations (Müller et al. 2004; Johnson et al. 2020). For example: *Given that the age of each fish is unknown but the age distribution in each loch can be estimated, what is the difference between loch A and loch B in the probability that a randomly sampled fish will survive beyond age* τ *?*
- The research question concerns survival beyond a target age that may differ between populations, as in population viability analysis (Eberhardt 2002), epidemiology (Ohm et al. 2018), or when age is quantified via biophysical measures rather than time (Ludwig and Smoke 1980; Jackson et al. 2003). For example: *In which of two allopatric populations is a randomly sampled individual more likely to survive beyond sexual maturity, considering that sexual maturity is reached at a different age in each population?*
- The research question is about an event occurring within (or outside) a certain age window. For example, since menopause before age 45 has been associated with all‐cause mortality (Muka et al. 2016), one might ask: *What is the probability that a female from population A experiences menopause before age 45, compared with a female from population B?*

We use a simulation study and two real‐world datasets to illustrate how to implement TAUS and how to interpret its output. We also compare its statistical robustness and ecological value against those of traditional approaches to survival analysis (i.e., Cox PH and parametric survival models).

### 1.1 Survival Analysis: An Overview

Survival analysis is relevant to many fields, including biology, medicine and engineering. In practice, survival analysis involves the study of the probability of death, and of how this probability changes over time or as a function of some explanatory variables (Emmert‐Streib and Dehmer 2019). This is usually modelled as follows:

Let T be a nonnegative random variable denoting time until death. Its density function $f\left(t\right)$ is defined by:

(1) $$f\left(t\right)=\lim_{\Delta t\to 0}\frac{P\left(t\leq T<t+\Delta t\right)}{\Delta t}$$

The cumulative distribution function $F\left(t\right)$ shows the probability that death happens before or exactly at time t:

(2) $$F\left(t\right)=\int_{0}^{t}f\left(u\right)\;\mathrm{du}=P\left(T\leq t\right)$$

From here, one can derive the survival function $S\left(t\right)$ which depicts the probability that death will happen after time t (i.e., probability of survival until t):

(3) $$S\left(t\right)=1-F\left(t\right)=P\left(T>t\right)$$

Lastly, we can derive the hazard function $h\left(t\right)$, which illustrates the probability of immediate death at time *t* conditional on survival up to that time:
(4)

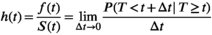




In practice, the true survival function of a population is unknown and must be estimated from data, which are often censored. For censored individuals, only partial information is available: their age at death is unknown, but it is known that death did not occur within a specific time interval. Statistical methods allow estimation of a population's survival function from such censored data. The Kaplan–Meier (KM) estimator provides a nonparametric estimate of $S\left(t\right)$ as a step function, properly accounting for censored observations. However, KM is limited to estimating survival curves within individual populations, and cannot be used to evaluate the effects of covariates on survival. For this purpose, the most common tools are the Cox and parametric approaches described above. Here, we present an alternative.

## 2 Methods

### 2.1 The TAUS Method

The full methodology for the development of TAUS is described in Supporting Information S1. A brief outline is offered here instead.

First, a formula is derived for the conditional survival function, which describes the probability of outliving some age τ conditional on being some age t (i.e., $P\left(T>\tau |T>t\right)$, Equation S5). The use of the conditional survival function instead of the normal survival function $S\left(t\right)$ (Equation 3) enables the study of age‐specific survival dynamics. Second, the conditional survival function is marginalised over the age distribution of the population in order to allow implementation when the current age of an individual is not known—which is often the case in ecological studies. The result of such marginalisation represents the probability that a randomly sampled individual of unknown age will outlive age τ, which we call $O_{\tau }$ (see Equation S6). This is followed by a comment on common practices and challenges when estimating the age distribution from data, where we point to some relevant literature on the topic.

The uncertainty around $O_{\tau }$ is reported in the form of 95% confidence intervals, which we calculate through the propagation of the uncertainty around the Kaplan‐ Meier estimator. In addition, we propose a statistical test that produces a *p*‐value for the assessment of the null hypothesis that two $O_{\tau }$ values, together with their 95% CI, are significantly different from each other.

### 2.2 Assumptions

The TAUS method does not assume proportional hazards or a parametric form for the survival distribution like Cox PH and parametric survival models. However, it does make some assumptions that are important to consider when applying it to real‐world data:

- The data are sufficient to estimate the survival function $S\left(t\right)$ across the range of t of interest: All metrics are derived ultimately from $S\left(t\right)$, so it is crucial that this function is well approximated. High sample sizes and long follow‐up periods would improve the model's precision significantly.
- The age distribution of the population can be known: Our method assumes that the age distribution $A\left(t\right)$ is a knowable (or at least, approximable) distribution. However, age structures of wild populations are rarely stable, and this has been argued theoretically (May 1974), and confirmed experimentally (Costantino et al. 1997) and observationally (Hoy et al. 2020).

### 2.3 Summary of Inputs and Outputs

The TAUS method takes a life table as input, which is the standard for survival analysis (Flynn 2012). It also requires that an approximation of the age distribution $A\left(t\right)$ be provided, although this can be estimated from $S\left(t\right)$ in the simplest case of stationary populations (see Supporting Information S1). The model outputs are summarised below:

- $P\left(T>\tau |T>t\right)$, the conditional survival matrix. This is a 2‐dimensional matrix where the element $\left(t,\tau \right)$ represents the probability that an individual of age t will outlive age τ. It is defined for all positive values of t and τ that are smaller than the largest age recorded in the study. This matrix is a function of both t (i.e., the age of the individual) and τ (i.e., the target age beyond which survival is of interest).
- $O_{\tau }\left(t\right)$, the probability of outliving age τ conditional on the current age t. This is equivalent to the τ‐th row of the conditional survival matrix, and is a function of t (i.e., the age of the individual).
- $O\left(\tau \right)$, the probability of outliving age τ when the age is unknown, as a function of τ. This function may look similar in shape to $S\left(t\right)$, but it is fundamentally different. While $S\left(t\right)$ describes the probability *at birth* of surviving until age t, $O\left(\tau \right)$ describes the probability *of a randomly sampled individual* of surviving until age τ. This is done by marginalising the conditional survival matrix over the t dimension using the age distribution $A\left(t\right)$ as a weighing function.
- $O_{\tau }$, the probability of outliving age τ when the age is unknown, for a particular value of τ. This can be understood as a specific case of $O\left(\tau \right)$, or, equivalently, as the area under the curve of $O_{\tau }\left(t\right)$ weighed by $A\left(t\right)$.
- Confidence intervals for all of the above (default is 95%, but can be customised).
- Pairwise statistical comparison of $O_{\tau }$ estimates, for any two populations and τ values of interest (i.e., different populations can have different values for τ).

### 2.4 Method Assessment Using Simulated and Real‐World Data

To evaluate the performance and practical utility of TAUS, we conducted one simulation study and downloaded two publicly available real‐world ecological datasets. We used these data to compare our method to the two most common approaches to survival analysis (Cox's PH model and parametric survival model).

The simulated dataset included survival data on two allopatric populations of a hypothetical species, hereafter referred to as Island A and Island B. Each island had a specific birth rate and baseline life expectancy. All individuals had a 50% chance of carrying a mutation, which affected their odds of survival in a complex way: for individuals with a low baseline life expectancy (< 15), the mutation increased their survival. For those with high baseline life expectancy (> 25), the mutation was beneficial in Island A and detrimental in Island B. Table 1 shows the exact processes and parameters used to simulate these data.

**TABLE 1:** Processes and parameters used to simulate the survival dataset.

Feature	Process	Island A	Island B	Description
Daily births	$P\left(\lambda \right)$	λ=7	λ=12	Poisson‐distributed daily births with island‐specific rate λ.
Baseline life expectancy	$\pi \cdot N\left(\mu_{1},\sigma_{1}\right)+\left(1-\pi \right)\cdot N\left(\mu_{2},\sigma_{2}\right)$	π=0.34 $\mu_{1}=10$ $\sigma_{1}=3$ $\mu_{2}=60$ $\sigma_{2}=20$	π=0.18 $\mu_{1}=10$ $\sigma_{1}=3$ $\mu_{2}=60$ $\sigma_{2}=20$	Bimodal Gaussian, shared means & SDs, mixture weight π varies by island.
Probability of mutation	Fixed value	p=0.5	p=0.5	Each individual carries the mutation with probability 50%.
Mutation effect on lifespan (if baseline < 15)	$N\left(\mu ,\sigma \right)$	μ=10 σ=5	μ=10 σ=5	Gaussian increase in life expectancy.
Mutation effect on lifespan (if baseline > 25)	$N\left(\mu ,\sigma \right)$	μ=35 σ=20	μ=−15 σ=5	Island‐specific effect; sign of μ determines increase or decrease.

The simulated dataset consisted of 191,399 individuals. Prior to any analysis, we artificially subsampled the data to achieve realistic conditions in terms of censoring, sample size and follow‐up period.

**FIGURE 1:**
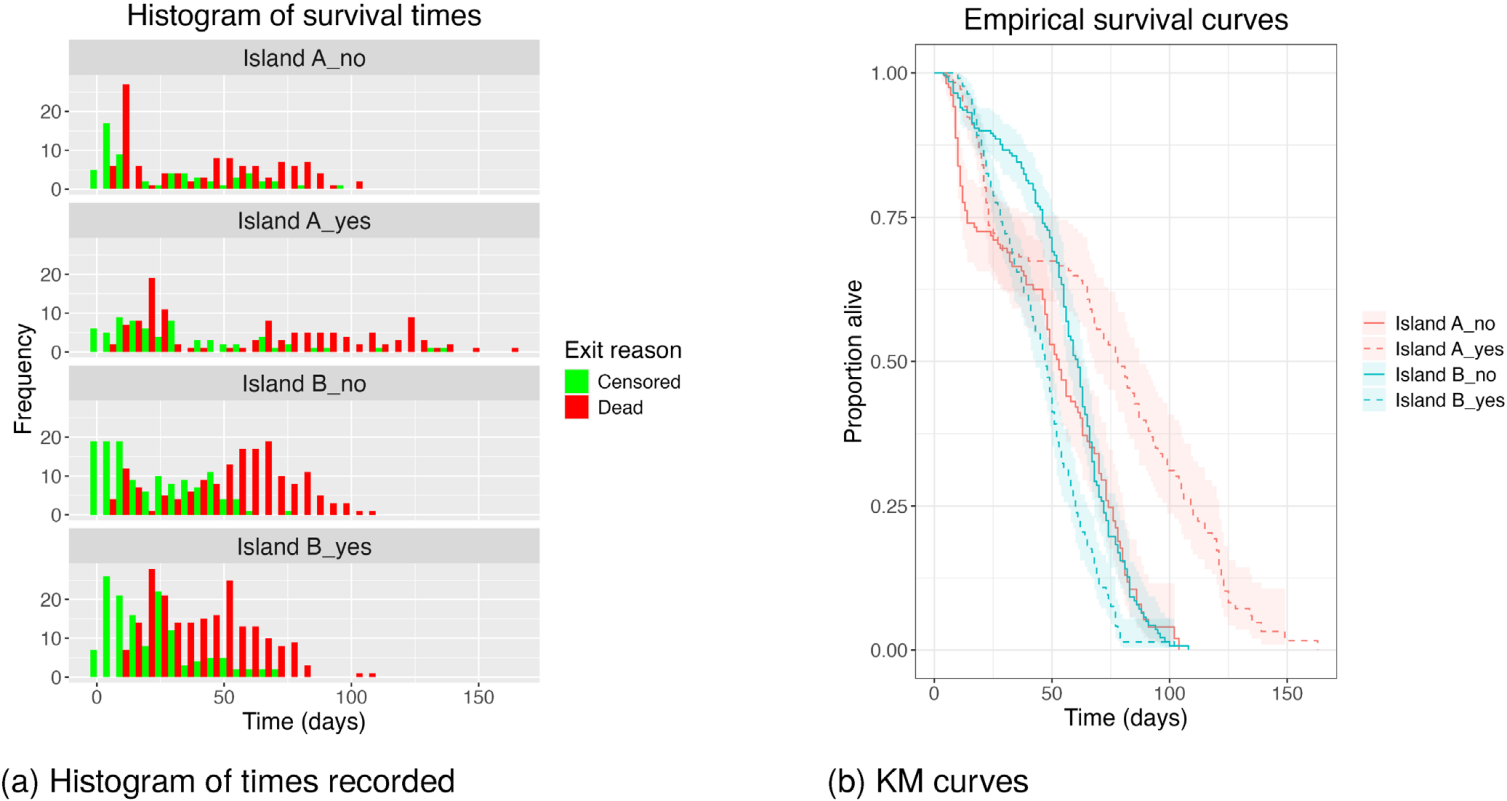
(a) Distribution of event times in the simulation study, aggregated by population (i.e., island: A/B) and genotype (i.e., mutation status: Yes/No). The x‐axis represents the age of the individuals, and the y‐axis represents the number of individuals at each age. Red bars represent individuals that were followed up until death and green bars represent individuals whose follow‐up ended for a different reason (right‐censored). (b) Kaplan–Meier (KM) curves for each subpopulation in the simulation study. The *x*‐axis represents time, and the *y*‐axis represents survival probability. The data are aggregated by population (i.e., island: A/B) and genotype (i.e., mutation status: Yes/No). Shaded bands represent the 95% CI.

Subsampling the dataset this way also enabled the assessment of a key assumption of our model, namely that the true population's age distribution $A\left(t\right)$ can be estimated from data (Figure 5). The subsampling was done as follows, and Figure 1 describes the final working dataset:

- *Censoring*: We randomly selected 40% of the individuals and truncated their recorded lifespan to a random value between 0 and their true age at death. These individuals were then marked as right‐censored in the dataset.
- *Sample size*: We randomly selected 1000 individuals and discarded the rest.
- *Follow‐up period*: The follow‐up period was set to 200 days, which aims to represent an appropriate experimental design given the lifespan of the simulated species. Individuals that were alive at the end of the follow‐up period were right‐censored at their age at that time.

This simulation aimed to resemble real‐world ecological survival data with credible features. In this scenario, ecologists are collecting data from two allopatric populations (e.g., bats) in a metapopulation context (e.g., an archipelago) where movement across subpopulations (e.g., islands) is possible. Of all subpopulations, only two are accessible to the ecologists (e.g., funding/logistic limitations). The hypothetical research aim is to describe the effect of a certain mutation on survival.

The two real‐world case studies illustrate how Cox PH, parametric survival modelling, and TAUS perform when applied to real datasets. One dataset has data on the survival of *Drosophila* flies from different countries (Klepsatel et al. 2013). The other one, on the survival of *Drosophila* flies reared at different temperatures (Gerken 2023). Both datasets are publicly available and were obtained from online repositories. The code used to simulate the data, and to analyse the three datasets is available in the R package TAUS (https://github.com/casasgomezuribarri/TAUS).

Cox models were fitted using the survival::coxph function in R. Parametric survival models were fitted using the flexsurv package in R. First, the most appropriate distribution was selected among all of those available in the package (i.e., Exponential, Log‐Normal, Log‐Logistic, Gamma, Gompertz, Weibull, Generalised Gamma and Generalised F). This was done by fitting a null model to the whole dataset with each distribution, and then selecting the best fit through a combination of formal goodness‐of‐fit metrics (AIC, Log‐likelihood and KS test) and visual inspection of the models' fit to the empirical survival, hazard and cumulative hazard functions. The TAUS analyses presented here can be reproduced using the TAUS R package (https://github.com/casasgomezuribarri/TAUS).

## 3 Results

### 3.1 Simulation Study

A simulated dataset was created as described in Section 2.4, aiming to resemble a realistic ecological scenario in terms of underlying processes, sample size, censoring and follow‐up period. The dataset consisted of survival data on two allopatric populations, where individuals could either carry or not carry a mutation that affected their survival in a complex, population‐specific manner.

#### 3.1.1 Cox PH

The Cox PH model was specified with the following covariates for survival times: mutation status, island and their interaction. As expected from the crossing KM curves (Figure 1b), the PH assumption was found to be violated (p<<0.05). Thus, this model was considered invalid and should not be used. However, with the purpose of output comparison, we ignore this and proceed to interpret the model's outputs (Table 2).

**TABLE 2:** Output of the Cox PH model applied to the simulated dataset.

Variable	HR (exp(*β*))	95% CI	*p*
Mutation (Yes)	0.38	0.29–0.51	4.3 × 10^−11^
Habitat (Island B)	0.84	0.66–1.1	0.15
Mutation (Yes) × Habitat (Island B)	4.2	3.0–6.0	2.2 × 10^−15^
Reference level: Habitat = Island A, Mutation = No			
Model statistics: n=1000, events = 607			
Concordance = 0.571 (SE = 0.014)			
Likelihood ratio test = 100.8 on 3 df, *p* < 2 × 10^−16^			
Wald test = 90 on 3 df, *p* < 2 × 10^−16^			
Score (logrank) test = 95.04 on 3 df, *p* < 2 × 10^−16^			

The reported Likelihood ratio, Wald and Score test (all with p<<0.05) agree on the good fit of the model. However, the Concordance index reveals a predictive performance (0.57) extremely close to random (0.5) (Longato et al. 2020). Altogether, these statistics suggest that, although the choice of explanatory variables was appropriate, the model was not able to accurately describe the survival dynamics of the system, which is consistent with the violation of the PH assumption onto which Cox PH models rely.

The hazard ratio for the variable *mutation* (HR=0.38, p<<0.05) indicates that, at any point in time, the immediate risk of death for individuals with the mutation from the reference island (Island A) is 38% of that for individuals from the reference level (island: A, mutation: No). The output also suggests that individuals from Island B with the reference mutation status (No mutation) have an immediate risk of death that is not significantly different from that of individuals from the reference level (island: A, mutation: No; HR=0.84, p=0.149). Lastly, the interaction term indicates that individuals from Island B with the mutation have an immediate risk of death that is, at all times, 0.38×0.84×4.2=1.34 times that of individuals from the reference level (p<<0.05).

#### 3.1.2 Parametric Survival

**FIGURE 2:**
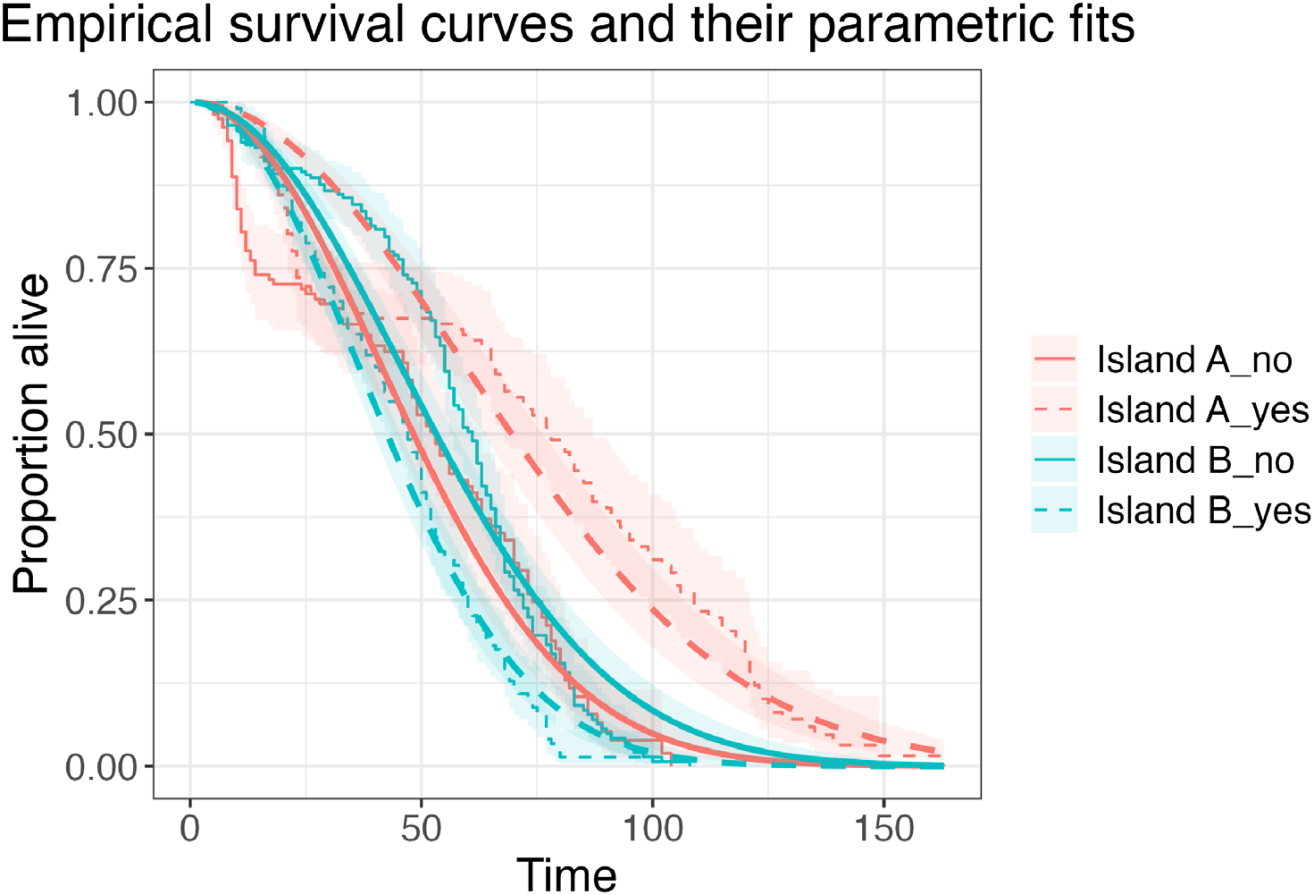
Empirical KM curves of each subpopulation in the simulation study (step curves), overlapped with their respective parametric fits (smooth curves). Data are aggregated by island (A: Red, B: Blue) and mutation (No: Solid lines, Yes: Dashed lines). Shaded regions represent the 95% confidence intervals.

The Weibull distribution was found to be the best fit available for the parametric survival analysis, which prompted an Accelerated Failure Time (AFT) model (Jackson et al. 2011). As with the Cox PH model, this was specified with the following covariates for survival times: mutation status, island and their interaction (Table 3, Figure 2).

**TABLE 3:** Output of the parametric survival model (Weibull, AFT) applied to the simulated dataset.

Variable	Estimate (*β*)	AFT factor (exp(*β*))	Std. error	Statistic	*p*
Shape (k)	2.0	NA	0.065	NA	NA
Scale (λ)	58.0	NA	2.7	NA	NA
Mutation (Yes)	0.36	1.4	0.065	5.6	2.3 × 10^−8^
Habitat (Island B)	0.097	1.1	0.061	1.6	0.11
Mutation (Yes) × Habitat (Island B)	−0.59	0.88	0.083	−7.1	1.6 × 10^−12^

The output (Table 3) lists the parameters for the baseline Weibull hazard function and how the specified covariates modify them. The shape parameter k≈2 is common to all groups, and indicates that the risk of death increases over time in a roughly linear manner (Incerti 2019). The scale parameter controls the stretching of the *x*‐axis (i.e., time), acting as a constant that sets the overall death rate (or, more precisely, the speed at which time is experienced). The coefficients reported by the model are the modifications to the scale parameter due to each explanatory variable (Jackson et al. 2011). The coefficient of the variable *mutation* indicates that individuals from the reference island (Island A) with the mutation experience time 1.4 times slower than the baseline group (island: A, mutation: No; p<<0.05), meaning that their lifespan is increased by 40%. Similar to the Cox model, the effect of island alone was not significant when the mutation status was at its reference level (No mutation; p=0.11). The interaction term indicates that individuals from Island B with the mutation experience time 0.88 times as fast as the baseline group, which means that their rate of death is increased by 12% (p<<0.05). This result is graphically presented in Figure 2.

This modelling approach provided a better description of the survival dynamics than the Cox PH model by explicitly accounting for the baseline hazard function, which removed the PH assumption (Cox and Reid 1994). However, the Weibull distribution was a poor fit to the empirical survival functions, which is illustrated by the general lack of shape overlap between the empirical and fitted curves, particularly at early times (Figure 2). Furthermore, the description of covariate effects on survival was limited to a single, time‐invariant value per covariate (i.e., the AFT factor).

#### 3.1.3 TAUS

**FIGURE 3:**
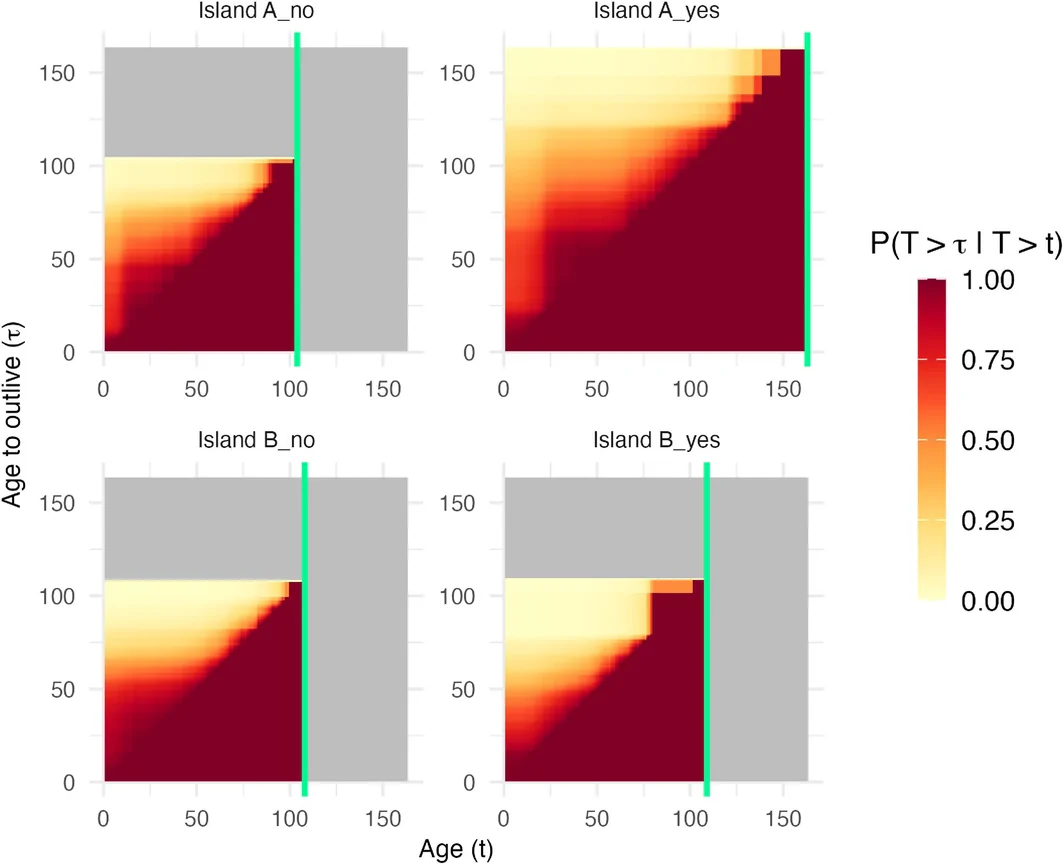
Each panel shows the conditional survival matrix for one of the four groups in the simulation study. The x‐axes represent the current age of the individuals (t), and the y‐axes represent the age beyond which survival is of interest (τ). The colour of each cell (x,y) represents the probability that an individual of age t=x will outlive age τ=y, that is, $P\left(T>\tau |T>t\right)$. Grey cells represent coordinates $\left(t,\tau \right)$ for which this probability is undefined (i.e., τ,t≥ maximum age observed in the group). The vertical green lines mark the time of the last death observed in each group.

The TAUS method is implemented in the R package TAUS (https://github.com/casasgomezuribarri/TAUS). The previous sections demonstrate that Cox PH and parametric survival models were limited in their ability to describe the simulated system. The Cox model was invalid, due to the violation of the PH assumption. The parametric model was valid, but its fit to the empirical data was debatable (the shape of the fitted curves did not match the shape of the empirical curves), and its description of covariate effects on survival was limited to a stretching/compressing parameter for this fit along the time dimension. TAUS, on the other hand, makes no assumptions about the hazard function, and relies entirely on the empirical survival function. For each population, it computes all possible conditional survival probabilities (i.e., $P\left(T>\tau |T>t\right)$ for all possible combinations of τ and t) and compiles them into a matrix. Figure 3 shows these as heatmaps.

**FIGURE 4:**
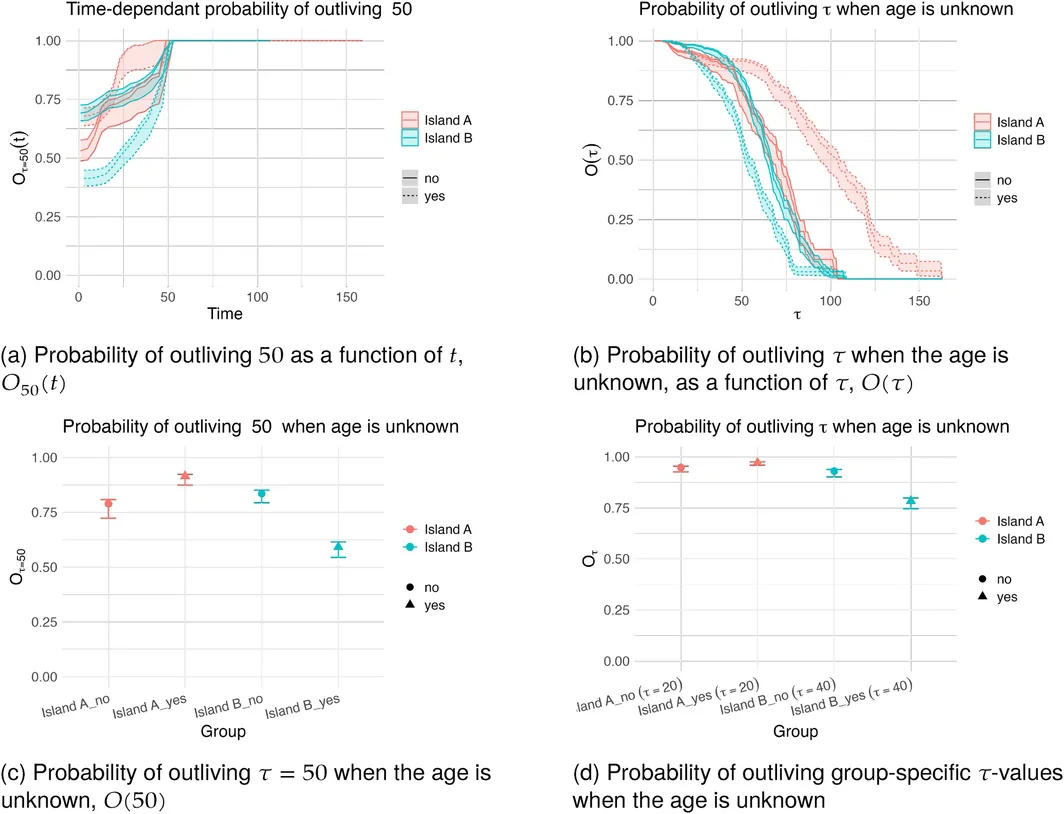
A summary of the possible outputs of the TAUS method. (a) The probability of outliving age τ=50 as a function of the current age t. (b) The probability of outliving τ when the age is unknown, as a function of τ. (c) The probability of outliving age τ=50 when the age is unknown. (d) The probability of outliving group‐specific ages (τ=20 for island A and τ=40 for island B) when the age is unknown. All panels include 95% CI limits and aggregate the data in four groups, according to island of origin (A/B) and mutation status (yes/no).

From these matrices, we can compute all outputs outlined in Section 3.3 (Figure 4).

Figure 4a shows that individuals with a mutation from island A have a consistently higher probability of outliving the arbitrary age of τ=50 than the other groups, regardless of their current age (i.e., highest value of $O_{50}\left(t\right)$, regardless of *t*). It also shows that individuals without the mutation have a similar age‐dependent probability of outliving τ=50 during most of their lives, regardless of their island of origin. As expected, the probability of outliving age 50 is 1 for any individual that is already 50 or older.

Figure 4b shows that this trend is true for almost any age τ to be outlived, not just τ=50. Individuals from island A with the mutation have the highest probability of outliving virtually any age. Individuals with the mutation from island B have the highest probability of outliving young ages, but their chances of surviving beyond any age greater than τ≈30 are the lowest in the study. Individuals without the mutation have similar chances of outliving most ages, but those from island A experience higher early life mortality.

Figure 4c shows that the probability of a randomly‐sampled individual (and thus, of unknown age) from island A of outliving the age of τ=50 is higher than for any other population. It also suggests that this probability is similar for individuals without the mutation, regardless of their island of origin. Lastly, a random individual with the mutation from island B would have the lowest chances of living beyond this age.

Figure 4d is similar, but in this case, the age beyond which survival is of interest is island‐specific. Arbitrarily, we chose τ=20 for island A and τ=40 for island B, although any value of τ can be chosen for any of the four populations. Figure 4d shows that having the mutation on island A has a positive effect on the chances of individuals surviving beyond τ=20, although this effect is non‐significant (Table 4). On the other hand, on island B, the effect of the mutation on chances of survival beyond τ=40 is negative and significant (Table 4).

**TABLE 4:** All pairwise comparisons of $O_{\tau }$ values (as shown in Figure 4d). For each group, the table shows the subpopulation, the value of τ (which is island‐specific), and $O_{\tau }$ (probability of living beyond τ when age is unknown). The final column shows the *p* of the KS test comparing both groups.

Population	τ	$O_{\tau }$	Population	τ	$O_{\tau }$	*p*
Island A_no	20	0.945	Island A_yes	20	0.968	0.159
Island A_no	20	0.945	Island B_no	40	0.923	0.785
Island A_no	20	0.945	Island B_yes	40	0.714	<0.001
Island A_yes	20	0.968	Island B_no	40	0.923	0.012
Island A_yes	20	0.968	Island B_yes	40	0.714	<0.001
Island B_no	40	0.923	Island B_yes	40	0.714	<0.001

Together, these figures illustrate the versatility of the output of TAUS. For example, inspection of Figure 4b suggests that, for individuals without the mutation—and regardless of island of origin—the probability of living beyond ages >50 is very similar. In contrast, those from island B have slightly higher chances of surviving beyond ages <50. It is also evident that the mutation is detrimental on island B while beneficial on island A. All of this is consistent with the processes used to simulate the data (Table 1).

Figures 4c,d showcase how the TAUS output can be used to compare survival probabilities across groups for specific ages of interest, which can be different for each group. This is particularly relevant in ecological settings where the chronological age at which something (e.g., metamorphosis) happens may vary across allopatric populations due to the specific conditions of their respective environments. Crucially, TAUS allows these comparisons to be statistically tested. Table 4 illustrates this for the comparisons shown in Figure 4d.

**FIGURE 5:**
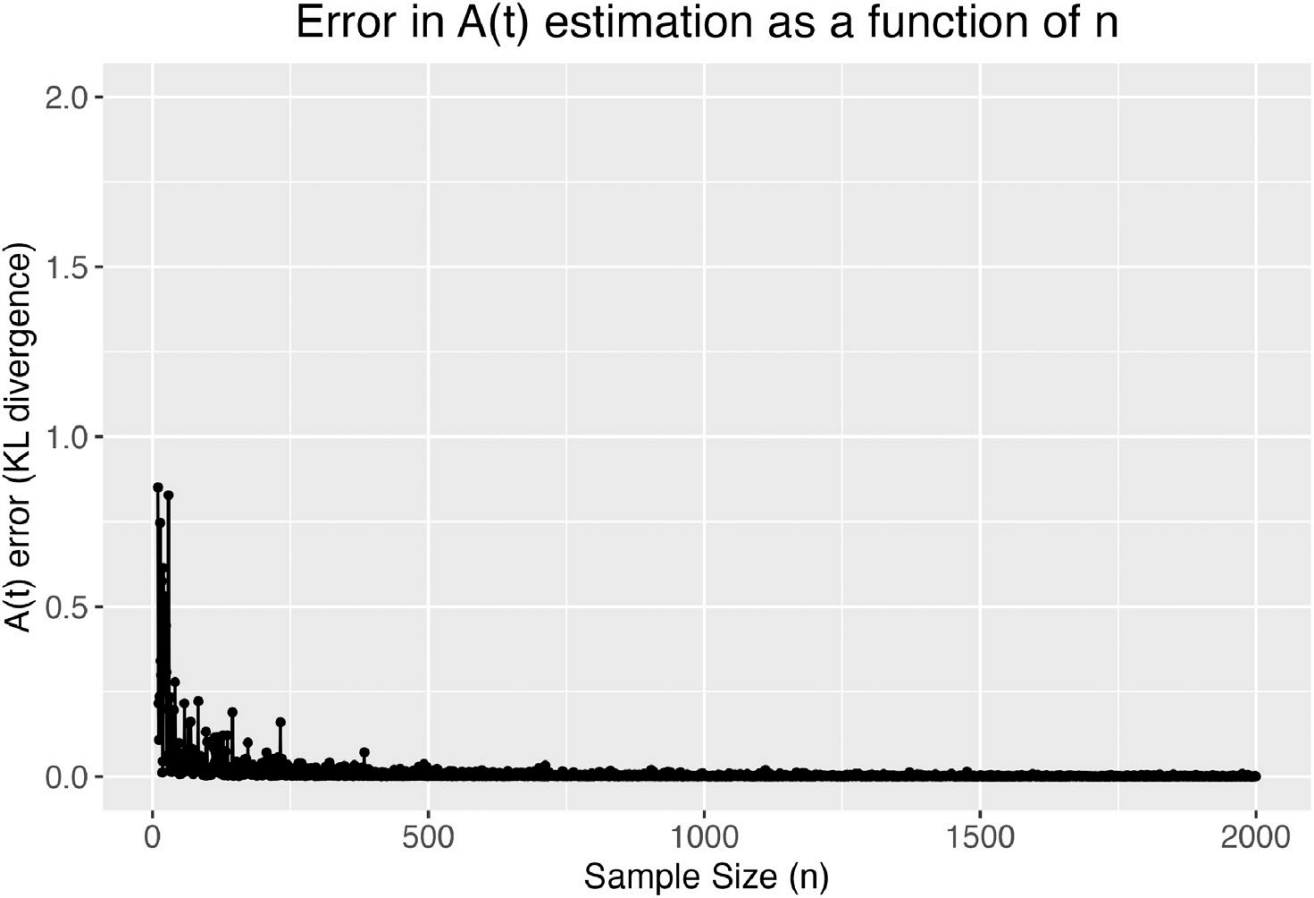
Power analysis based on the accuracy of the estimation of the true age distribution ($A\left(t\right)$), computed as the KL divergence between the estimated and ground truth distributions, as a function of sample size.

The strongest assumption of the method is that the survival function $S\left(t\right)$ (which is proportional to the age distribution $A\left(t\right)$ for stable populations—no growth, no migration) is well estimated. In real‐world cases, this is often impossible to assess. However, for our simulated dataset, we can compute the age distribution using different subsample sizes, and calculate the KL divergence to compare these estimates with the true age distribution (as computed with the whole dataset, n=191,399). Figure 5 shows that, beyond a sample size of n≈1000, the estimation of the true age distribution does not improve much with larger sample sizes. For this specific case study, a sample size under n≈500 would have likely not been sufficient.

### 3.2 Example 1: Flies From Different Countries

**FIGURE 6:**
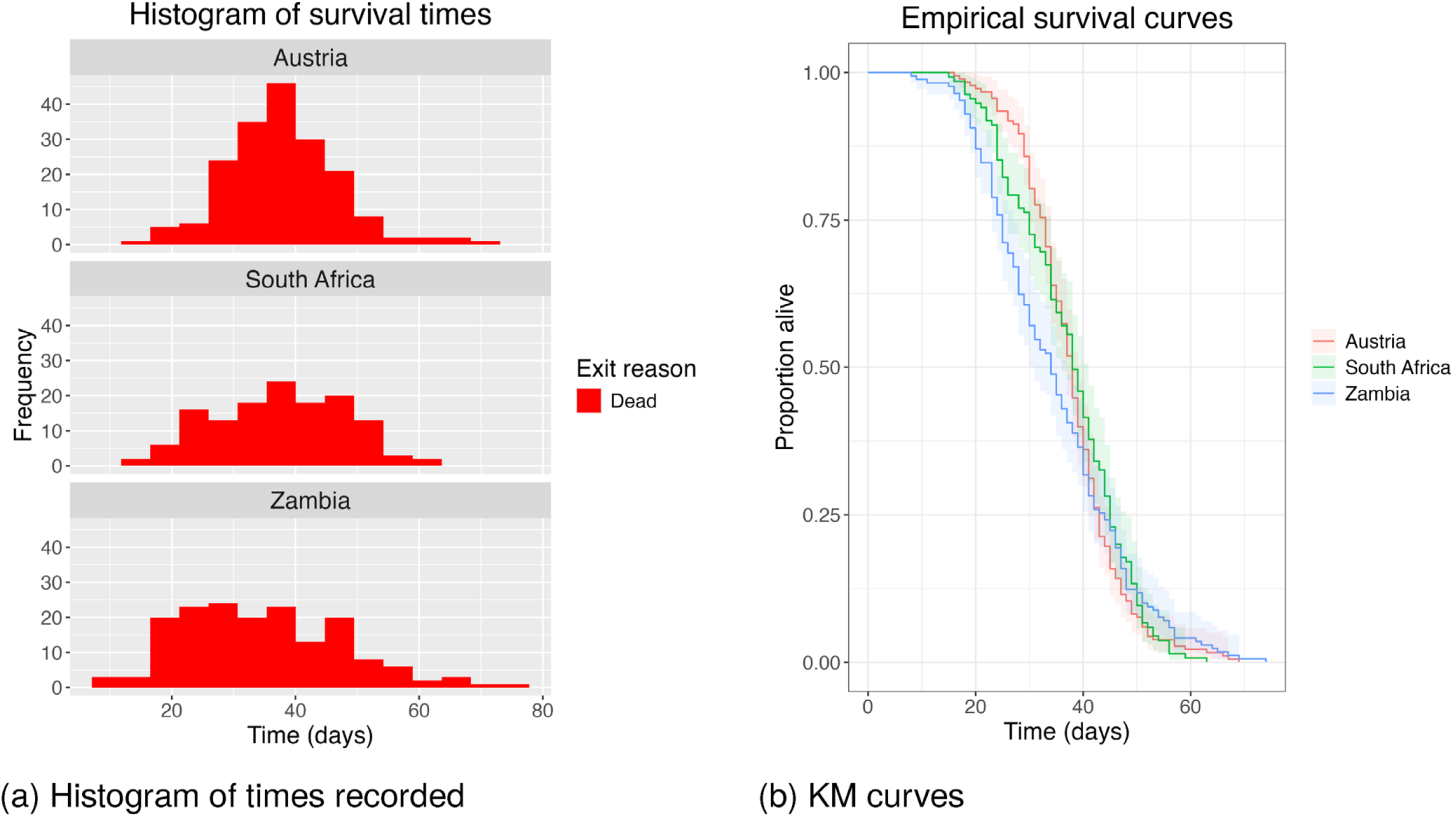
(a) Distribution of event times of the three populations in the study. The x‐axis represents the age of the individuals, and the y‐axis represents the number of individuals at each age. Red bars represent individuals followed up until death and green bars represent individuals whose follow‐up ended for a different reason (censored). Note the absence of censored individuals in this study. (b) Kaplan–Meier curves for each population in the study. The x‐axis represents time, while the y‐axis represents survival probability.

The dataset analysed here (publicly available (Klepsatel et al. 2013)) contains data on fly survival, origin, fecundity, fertility and physiology. In order to keep this example focused, some variables were removed so that only one explanatory variable was considered. After pre‐processing, the dataset contained survival data on wild‐caught *Drosophila* flies from three different countries (Figure 6).

The survival dynamics of all three populations were comparable, but with some differences. Austrian flies had the lowest mortality rates at early and late ages, but showed the highest mortality rates at mid‐ages. Zambian flies had the highest mortality rate at early ages, but they also recorded the longest lifespans. South African flies lay somewhere in the middle. One of the key features of this dataset is the lack of censored individuals: all flies were observed until death (Figure 6).

#### 3.2.1 Cox PH

The Cox model was specified with country of origin as the only covariate for the survival times. The model was found to be invalid due to the violation of the PH assumption (p<<0.05). Again, this was expected from the crossing of the KM curves in Figure 6b. The output of this model is reported in Table 5 for completion purposes. However, due to its invalidity, this model was not considered and its output is not be discussed.

**TABLE 5:** Output of the Cox PH model applied to the dataset from Example 1: Flies from different countries.

Variable	HR (exp(*β*))	95% CI	*p*
Population (South Africa)	0.95	0.77–1.2	0.67
Population (Zambia)	1.0	0.84–1.3	0.70
Reference level: Population = Austria			
Model statistics: n=488, events = 488			
Concordance = 0.541 (SE = 0.016)			
Likelihood ratio test = 0.54 on 2 df, p=0.8			
Wald test = 0.54 on 2 df, p=0.8			
Score (logrank) test = 0.54 on 2 df, p=0.8			

#### 3.2.2 Parametric Survival

**FIGURE 7:**
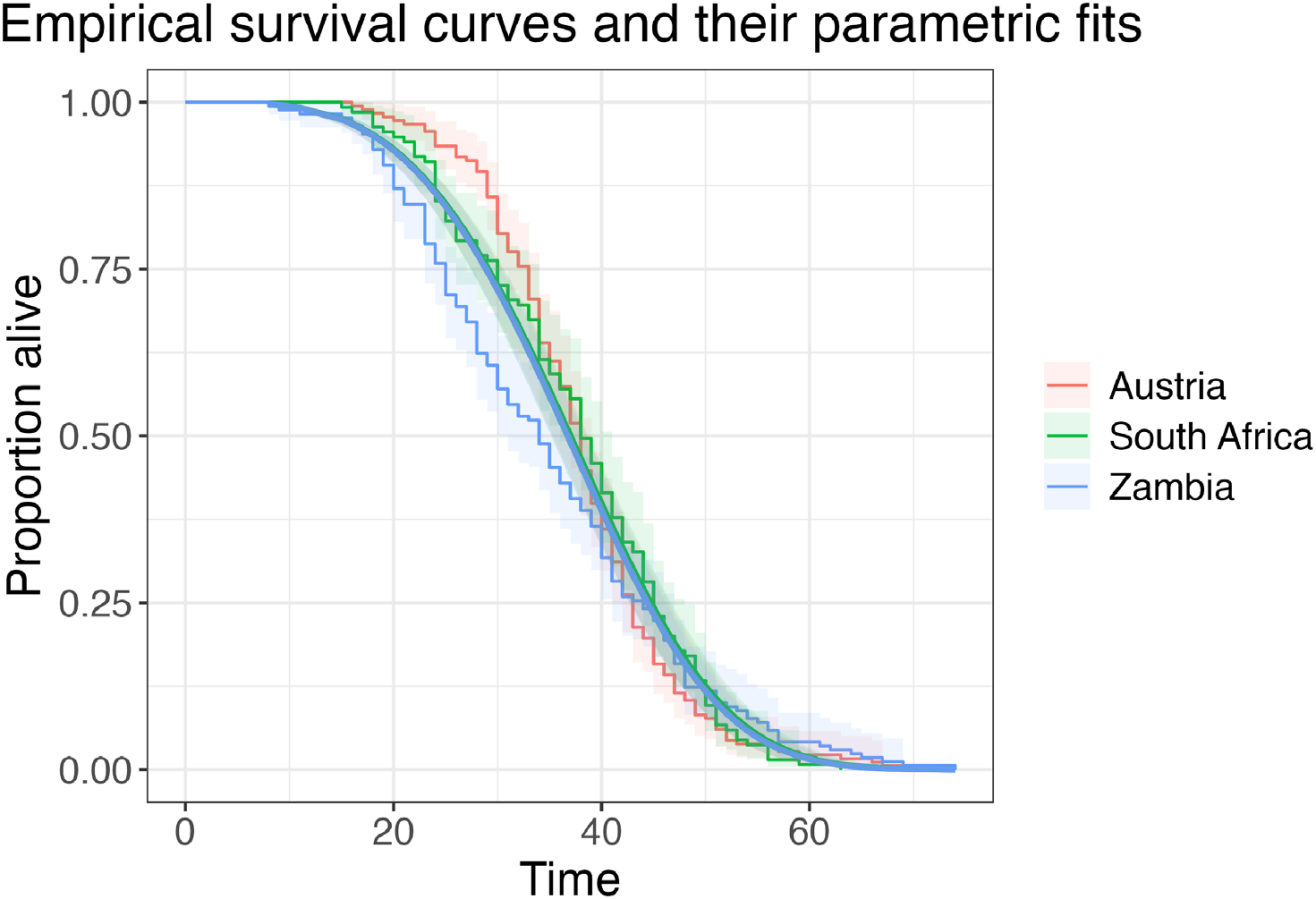
KM curves of each population in the study (step curves), overlapped with their respective parametric fits (smooth curves). Confidence intervals (95%) are shown as shaded regions.

The parametric survival model was fitted using the Weibull distribution, which again prompted an AFT model. It was specified with country of origin as the sole explanatory variable. The model estimated survival to be identical across populations (Table 6, Figure 7).

**TABLE 6:** Output of the parametric survival model (Weibull, AFT) applied to the dataset from Example 1: Flies from different countries.

Variable	Estimate (*β*)	AFT factor (exp(*β*))	Std. error	Statistic	*p*
Shape (k)	3.7	NA	0.13	NA	NA
Scale (λ)	41	NA	0.83	NA	NA
Population (South Africa)	0.0042	1.0	0.031	0.14	0.89
Population (Zambia)	−0.0030	1.0	0.029	−0.10	0.91

#### 3.2.3 TAUS

**FIGURE 8:**
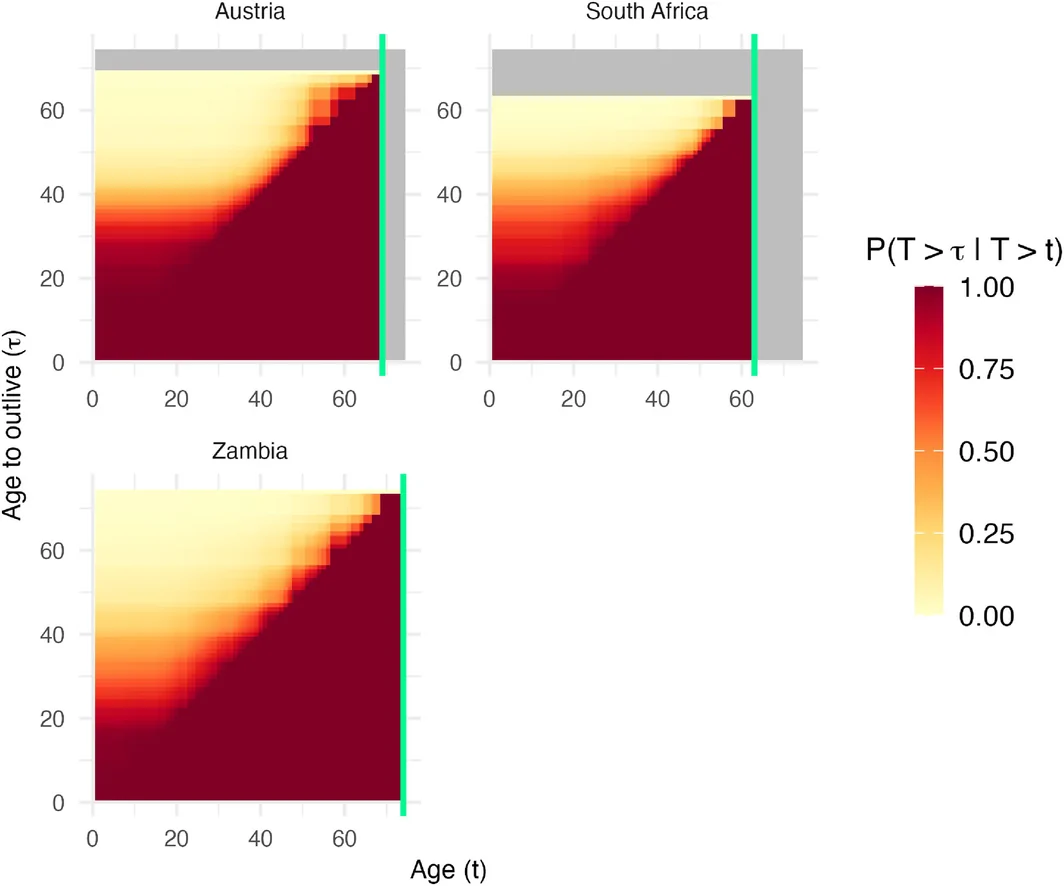
Each panel shows the conditional survival matrix for one of the groups in the study. The x‐axes represent the current age of the individuals (t), while the y‐axes represent the age beyond which survival is of interest (τ). The colour of each cell (x,y) represents the probability that an individual of age t=x will outlive age τ=y, that is, $P\left(T>\tau |T>t\right)$. Grey cells represent coordinates $\left(t,\tau \right)$ for which this probability is undefined (i.e., τ,t≥ maximum age observed in the group). The vertical green lines mark the time of the last death observed in each group.

Figure 8 shows the conditional survival matrices for this dataset (aggregated by country), from which all outputs of the TAUS method are calculated.

**FIGURE 9:**
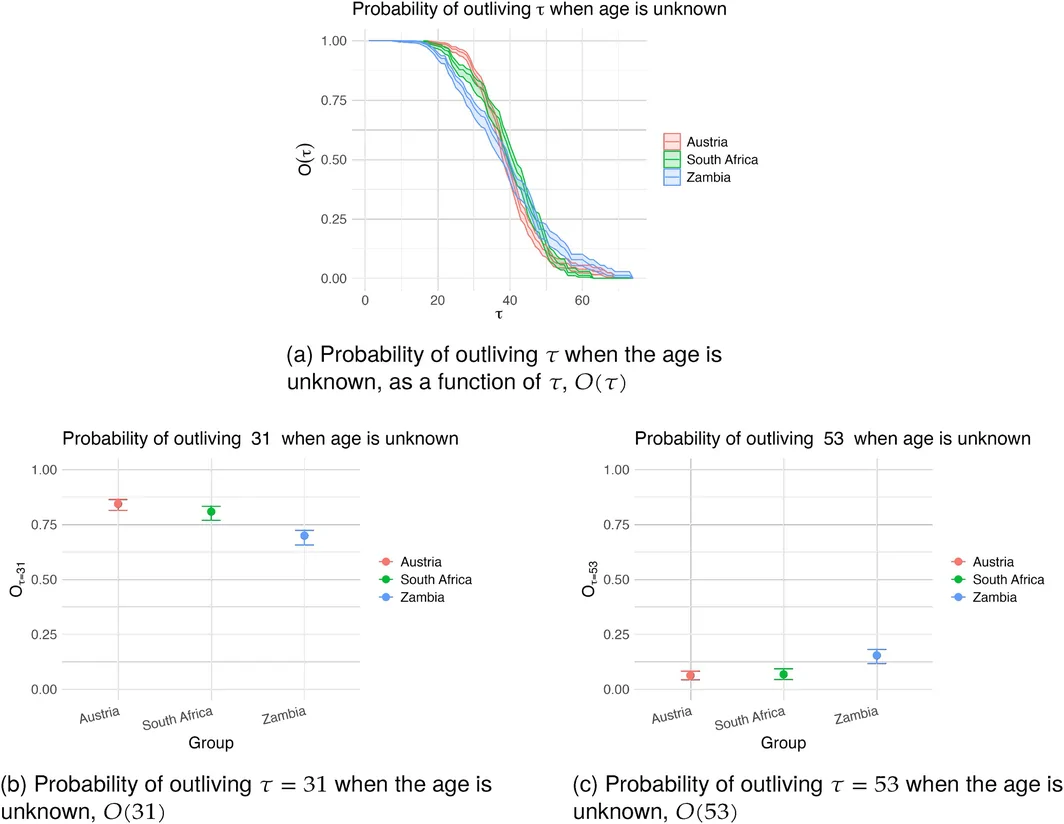
Outputs of the TAUS method describing the survival dynamics of flies from Austria (red), South Africa (blue) and Zambia (green). (a) The probability of outliving age τ as a function of τ of a randomly sampled individual of unknown age. (b) The probability of outliving age τ=31 of a randomly sampled individual of unknown age. (c) The probability of outliving age τ=53 of a randomly sampled individual of unknown age. Shaded region (a) and error bars (b,c) show the 95% CI bounds.

From the survival matrices, we can compute the $O_{\tau }$ curves (i.e., the probability of living beyond the age of τ for a randomly sampled individual of unknown age, as a function of τ—Figure 9a). These show that Zambian flies are less likely to outlive young ages (<40 days), and more likely to outlive old ages (>50 days) than the other groups. This suggests that Zambian flies experience a higher early‐life mortality rate, but that past a certain age, their mortality is lower than in the other populations, which is consistent with their true survival dynamics (Figure 6a).

Indeed, Figures 9b,c show that, although a randomly‐sampled fly from the Zambian population is less likely to survive age 31 than a counterpart from South Africa or Austria, it is more likely to survive age 53. The statistical significance of these comparisons is shown in Table 7. The other models (Cox PH and parametric) were not able to capture these differences (Tables 5 and 6, Figure 7).

**TABLE 7:** Pairwise comparisons of $O\left(\tau \right)$ for two values of τ.

Comparison		$O\left(\tau =31\right)$		$O\left(\tau =53\right)$	
Group 1	Group 2	*p*	Reject $H_{0}$?	*p*	Reject $H_{0}$?
Austria	South Africa	0.788	No	1.000	No
Austria	Zambia	0.002	Yes	0.015	Yes
South Africa	Zambia	0.012	Yes	0.051	No?

### 3.3 Example 2: Flies at Different Temperatures

This implementation example is provided as a supplementary material (Supporting Information S2). The example uses another publicly available dataset on *Drosophila* survival (Gerken 2023) to compare the TAUS method to the Cox PH and parametric survival models following the same steps as the example above. This dataset contains data on fly survival at different temperatures, and the distribution of times at death suggests that temperature strongly affected fly survival. However, in all treatments there was a small fraction of flies that lived extremely long lives and remained alive at the end of the experiment, and this was true for all temperature treatments (Figure S2). This example is interesting because of the differences in survivorship bias between treatments. In a population with high early‐life mortality, long‐lived individuals make up a larger proportion of the sampleable population, and therefore the probability of a randomly sampled individual outliving an old age is higher. Failing to capture this feature in a survival system can have important implications in ecological studies, especially in epidemiological settings where long‐lived individuals may contribute disproportionatelly to disease transmission.

In this example, the PH assumption is shown not to hold true, and the best parametric fit (Log‐logistic) wrongly predicted a negative association between temperature and the proportion of long‐lived individuals (Figure S3, Table S2). In contrast, TAUS correctly captured that high temperatures increased early life mortality while also increasing the chances of outliving old ages (Figure S5). This results highlight the importance of incorporating survivoship bias in survival analysis.

## 4 Discussion

We have developed a new method for survival analysis, TAUS, and demonstrated that it may often be better suited to ecological survival data than the most commonly used approaches, Cox PH and parametric survival models. The reason for this is two‐fold. First, TAUS is free from the modelling constraints inherent to standard survival models. Second, its output is more informative, enabling detailed examination of age‐specific survival probabilities.

The TAUS method does not assume proportional hazards nor does it parameterise the baseline survival function, which are common issues when applying Cox PH and parametric survival models to ecological datasets. Indeed, the PH assumption did not hold for any of the examples discussed here (simulated or real). The parametric assumption (i.e., that there exists a distribution that describes the fundamental survival dynamics of the population(s) under study) is challenging to confirm or reject beyond visual inspection of the fit under consideration. Accepting this limitation of assumption interrogation, the best fit available was selected for each example.

Noteworthily, the best fit for the two real‐world examples (which included data from the same genus *Drosophila*) was different (Weibull for Example 1, Log‐logistic for Example 2). If a parametric distribution exists that fundamentally describes the survival dynamics of *Drosophila*, it would be expected that this distribution be the same across experiments. If the opposite is to be accepted as true—that is, that different experiments can yield different best‐fitting distributions—this would suggest that no such universal distribution exists. In that case, choosing a parametric instance to describe and compare survival across experimental groups becomes somewhat arbitrary and overfitting to the specific case at hand. This is not to imply that the analysis is invalid, but rather that its generalisability is limited.

The output of the TAUS method is also more informative than the outputs of the Cox and parametric models, allowing for a richer analysis of survival data, as detailed in Section 3.3. Cox and parametric models, when epistemologically justifiable, offer relative outputs of one of two types: Proportional Hazards (PH) or Accelerated Failure Time (AFT). PH models estimate the hazard ratio (HR) between groups, which can be interpreted as ‘the immediate risk of death in population A is HR times that of population B’. The AFT factor, on the other hand, represents a dilation/contraction constant for the time dimension. Thus, its interpretation is of the type ‘Population A ages AFTfactor times as fast as population B’. Both interpretations reduce survival effects to a single factor change, either in risk or in time. This can be, and certainly is, useful in some contexts; but it can also miss important aspects of the survival dynamics. In contrast, TAUS returns the probability of outliving τ, where τ is some specific age over which survival is of interest. It also provides a framework for the statistical comparison of this probability across populations. Crucially, the value of τ can be chosen to be different for each population under analysis. This makes the method especially relevant to ecological systems, where the populations under comparison often have different ageing rates, and therefore the meaning of τ in terms of biological age may differ across them. Another use case can be found in vector‐parasite systems. In a context where two allopatric vector populations follow different survival dynamics and show different parasite incubation periods, TAUS has the potential of answering the question *In which population are individuals more likely to survive long enough to become infectious?*. Cox PH and parametric models are not suited to address this question.

The probability of outliving τ is initially calculated as conditional on the current age t of the individual, allowing for fine analysis of age‐dependent survival probabilities whenever appropriate. However, the model also allows the collapsing of these probabilities over the t dimension, effectively producing a metric for the probability of outliving τ when the age of the individual is unknown. This is especially useful in ecological settings, where the age of sampled wild individuals can be challenging to ascertain.

It should be noted that Equation S5 is mathematically equivalent to the KM‐sampling method used in recent work on conformalised survival distributions (Qi et al. 2024). Both methods apply the same conditional survival logic, but in a somewhat reverse sense: Qi et al. (2024) calculate $P\left(T>t|T>c\right)$ for all t>c and known c to decensor data for model training, whereas TAUS computes $P\left(T>\tau |T>t\right)$ for a fixed τ and unknown t (marginalising over $A\left(t\right)$), yielding a population‐level survival metric that corrects for the survivorship bias of the sampling process. Comparison of our figure S1 with their figure 3 (Qi et al. 2024) illustrates that these are two mirrored applications of the same logic.

When plotting the $O\left(\tau \right)$ curves (Figures 4b and 9a, Figure S5a), the result can look similar to survival curves, but it is fundamentally different. While survival curves show the probability *at birth* of surviving beyond τ, the $O\left(\tau \right)$ curves show this probability *at the age of sampling*. As the probability of sampling older individuals increases (e.g., in the presence of high early‐life mortality), the $O\left(\tau \right)$ curves diverge further from the survival curves (see for example Figure S5a). On the other hand, this divergence shrinks in cases where individuals are most likely to be sampled at young ages. This divergence is entirely due to the survivorship bias, which is the well‐known logical error of concentrating analysis on samples that have passed through a selection process (in this case, being alive at the time of sampling). Despite its semantic negative connotations due to words like *bias* and *error*, this is a desirable feature in survival analysis: we are only interested in the survival of individuals that are alive.

As mentioned in Section 3.2, TAUS relies heavily on a good description of the age distribution of the population of interest. The simulation study presented here enabled the assessment of this assumption, but this is rarely possible in real‐world cases. The age distribution of a biological system is usually subject to continuous and sometimes radical change. As a result, when applying this model, reasonable effort should be put into describing the relevant age distributions as accurately as possible. The age distribution can be empirically estimated from fertility and mortality data (Lotka 1937; Caswell 2001; Howard 2026; Inaba 2017), or from mark‐recapture experiments (Müller et al. 2004, 2007). In addition, the collection of data on age at death for the longest‐lived individuals greatly facilitates the appropriate integration of the survivorship bias. One use case that can be of particular interest is when two populations have identical $S\left(t\right)$ but different $A\left(t\right)$—for example immediately after a colonisation, population control, or extreme weather event. While traditional methods that rely solely on $S\left(t\right)$ would be unable to distinguish between these two populations, TAUS would appropriately account for the differential effect of survivorship bias on both populations, giving a unique insight of the differences in survival expectations of individuals encountered from either one.

Survival analysis has traditionally been designed to investigate clinical data, where sample size is a common limitation, but information about each point in the sample is abundant. In ecological datasets the case is often the opposite: acquiring large sample sizes tends to be easier than ensuring a wealth of information about each point. In some cases, even the age of the individuals is unknown. The TAUS method is designed to be fit for purpose in this context, enabling the analysis of survival data with large sample sizes and little information per individual, and facilitating lifespan predictions on new data where the age of individuals is unknown. This is only possible because the metric for lifespan used is the probability of outliving a specific age, rather than the typical survival time (e.g., median), which is often the case.

In summary, TAUS offers a flexible and ecologically grounded alternative to standard survival models by avoiding assumptions that are difficult to justify in natural systems and by producing absolute, biologically meaningful survival estimates. By aligning the statistical output with the realities of ecological processes and data collection, TAUS opens new opportunities for comparing survival across populations. We anticipate that this approach will complement existing methods and provide researchers with a principled tool for studying survival in complex ecological settings.

## Author Contributions

**Iván Casas Gomez‐Uribarri:** conceptualization (lead), data curation (lead), formal analysis (lead), investigation (lead), methodology (lead), software (lead), validation (lead), visualization (lead), writing – original draft (lead), writing – review and editing (equal). **Simon A. Babayan:** conceptualization (equal), funding acquisition (supporting), methodology (equal), resources (equal), software (equal), supervision (equal), writing – review and editing (equal). **Fredros Okumu:** conceptualization (supporting), funding acquisition (equal), methodology (supporting), supervision (supporting). **Francesco Baldini:** conceptualization (equal), funding acquisition (lead), methodology (equal), project administration (lead), resources (lead), supervision (lead), writing – review and editing (lead). **Mauro Pazmiño Betancourth:** conceptualization (equal), funding acquisition (supporting), investigation (equal), methodology (equal), resources (equal), supervision (equal), writing – review and editing (equal).

## Funding

The Bill and Melinda Gates Foundation (INV‐003079). F.B. and M.P.B. were supported by the Academy of Medical Sciences' Springboard Award (ref: SBF007/100094).

## Conflicts of Interest

The authors declare no conflicts of interest.

## Supporting information


**Figure S1:** Estimation of $O_{\tau }$ for the arbitrary case of τ=15 (green dashed line). It shows the conditional survival $P\left(T>\tau |T>t\right)$ (black line, 95% CI in grey), the age distribution $A\left(t\right)$ (blue histogram), the *t*‐wise evaluation of $P\left(T>\tau |T>t\right)\cdot A\left(t\right)$ (red line) and the value of $O_{\tau }=\sum_{t=1}^{\infty }P\left(T>\tau |T>t\right)\cdot A\left(t\right)$ (red area; in practice the sum is over $t=1,\ldots ,t_{\max}$).
**Figure S2:** Example 2—data description. (a) Distribution of event times of the five populations in the study. The x‐axis represents the age of the individuals, while the y‐axis represents the number of individuals at each age. Red bars represent individuals that were followed up until death and green bars represent individuals whose follow‐up ended for a different reason (censored), in this case due to the end of the experiment. (b) Kaplan–Meier curves for each population in the study. The *x*‐axis represents time, and the *y*‐axis represents survival probability at birth.
**Table S1:** Output of the Cox PH model applied to the dataset from Example 3: Flies at different temperatures.
**Figure S3:** KM curves of each population in the study (step curves), overlapped with their respective parametric fits (smooth curves).
**Table S2:** Output of parametric survival model fitted to the data in Example 2. The table shows the effect on the Log‐logistic's distribution location parameter (the scale parameter) of each temperature group against the reference group (14°C). This table is a modified version of the output that can be obtained calling the R function flexsurv::tidy() on the fitted model object.
**Figure S4:** Each panel shows the conditional survival matrix for one of the groups. The x‐axes represent the current age of the individuals (t), while the y‐axes represent the age beyond which survival is of interest (τ). The colour of each cell (x,y) represents the probability that an individual of age t=x will outlive age τ=y, that is, $P\left(T>\tau |T>t\right)$. Grey cells represent coordinates $\left(t,\tau \right)$ for which this probability is undefined (i.e., τ,t≥ maximum age observed in the group). The vertical green lines mark the time of the last death observed in each group.
**Figure S5:** Outputs of the TAUS method describing the survival dynamics of flies reared at different temperatures. (a) The probability of outliving age τ as a function of τ of a randomly sampled individual of unknown age. (b) The probability of outliving age τ=43 of a randomly sampled individual of unknown age. (c) The probability of outliving age τ=125 of a randomly sampled individual of unknown age. Shaded region (a) and error bars (b, c) show the 95% CI bounds.
**Table S3:** Pairwise comparisons of $O\left(\tau \right)$.

## Data Availability

The data and code necessary to reproduce our analysis are available at (https://github.com/casasgomezuribarri/TAUS).
