# Supplementary material for: TAUS: Target‐Age Unified Survival. Survival Analysis Without Assuming Proportional Hazards or Parameterising the Survival Function
Source: Ecol Evol. 2026 Sep 7;16(9):e74034. doi: 10.1002/ece3.74034 (PMC13550012; doi:10.1002/ece3.74034)
Supplement: Supplementary file 1 [file ECE3-16-e74034-s001.pdf]

Supporting Information: Target-Age Unified Survival (TAUS)

Iván Casas Gomez-Uribarri[1,*], Simon A. Babayan[1,†], Fredros Okumu[1,2,†], Francesco Baldini[1,2,†], and Mauro Pazmiño Betancourth[1,*,†]

[1]School of Biodiversity, One Health & Veterinary Medicine, University of Glasgow, Glasgow, UK
[2]Ifakara Health Institute, Ifakara, Morogoro region, Tanzania
[*]Corresponding authors: 2332990C@student.gla.ac.uk, Mauro.Pazmino@glasgow.ac.uk
[†]These authors contributed equally to the supervision of this work.

This Supporting Information accompanies the main article. Figure, table, equation, and section numbers are prefixed with S.

S1. DETAILED METHODOLOGY

Conditional survival

We derive a formula for the probability that an individual of age t will outlive age τ. Let T denote the time at death (a nonnegative random variable). Applying Bayes' theorem, we have:

$$P(T > \tau \mid T > t) = \frac{P(T > \tau) \cdot P(T > t \mid T > \tau)}{P(T > t)} \tag{S1}$$

Now, applying main-text Equation (1), we arrive at the following:

$$P(T > \tau \mid T > t) = \frac{S(\tau)}{S(t)} \cdot P(T > t \mid T > \tau) \tag{S2}$$

where $S(t)$ and $S(\tau)$ can be estimated from data using a standard KM model. We can structure the calculation of $P(T > t \mid T > \tau)$ in two logical cases:

The case when $t < \tau$

In this case, the individual's current age t is less than the age of interest τ. If an individual has outlived τ, it is certain that it has outlived t. This leads to the following conditional survival formula, substituting $P(T > t \mid T > \tau) = 1$ into Equation S2:

$$P(T > \tau \mid T > t) = \frac{S(\tau)}{S(t)} \tag{S3}$$

The case when $t \geq \tau$

Following the same logic, when $t \geq \tau$,

$$P(T > \tau \mid T > t) = 1 \tag{S4}$$

(i.e. it *has happened already*). Therefore, the probability of survival to age τ conditional on survival to age t can be formulated as follows:

$$P(T > \tau \mid T > t) = \begin{cases} \frac{S(\tau)}{S(t)}, & t < \tau \\ 1, & t \geq \tau \end{cases} \tag{S5}$$

It should be noted that the conditional survival space is defined for all values of t and τ that are smaller than the largest age recorded in the study.

The conditional survival function $P(T > \tau \mid T > t)$ (Equation S5) describes the probability that an individual of age t will outlive age τ. This contrasts with the traditional survival function $S(t)$, which is the specific case when $t = 0$ (i.e. survival probabilities as computed at birth). Indeed, if we set $t = 0$, we get $P(T > \tau \mid T > 0) = P(T > \tau) = S(\tau)$ (see main-text Equation (1)).

Probability of outliving age τ (O_τ)

To compare conditional survival across groups, variation must be considered along two dimensions: the age of the individual (t), and the age beyond which survival is of interest (τ). This makes the comparison of conditional survival more complex than the comparison of other traditional survival functions, which vary only along one dimension.

In order to simplify this comparison for practical applications, we propose that a choice of (one or a few values for) τ be made by the analyst. We also propose that the conditional survival $P(T > \tau \mid T > t)$ be collapsed over the t dimension by considering the distribution of t (i.e. the age structure of the population). Hence, O_τ can be defined as the probability that a randomly sampled individual of unknown age will outlive age τ. By the law of total probability, this is the expectation of $P(T > \tau \mid T > t)$ over the age distribution: $O_\tau = \mathbb{E}_{A(t)}[P(T > \tau \mid T > t)]$. Treating time discretely, this metric can be calculated as follows:

$$O_\tau = \sum_{t=1}^{t_{\max}} P(T > \tau \mid T > t) \cdot A(t) \tag{S6}$$

where $A(t)$ is the proportion of individuals in a population with current age t, also known as the age distribution of the population. Figure S1 illustrates how this metric is calculated for the arbitrary case of $\tau = 15$: O_τ equals the area under the red curve, which is the t-wise product of $P(T > \tau \mid T > t)$ (black line) and $A(t)$ (blue bars). Section S1.3 discusses different approaches to estimating $A(t)$, and Section S1.4 illustrates how uncertainty is propagated to provide a confidence interval around O_τ.

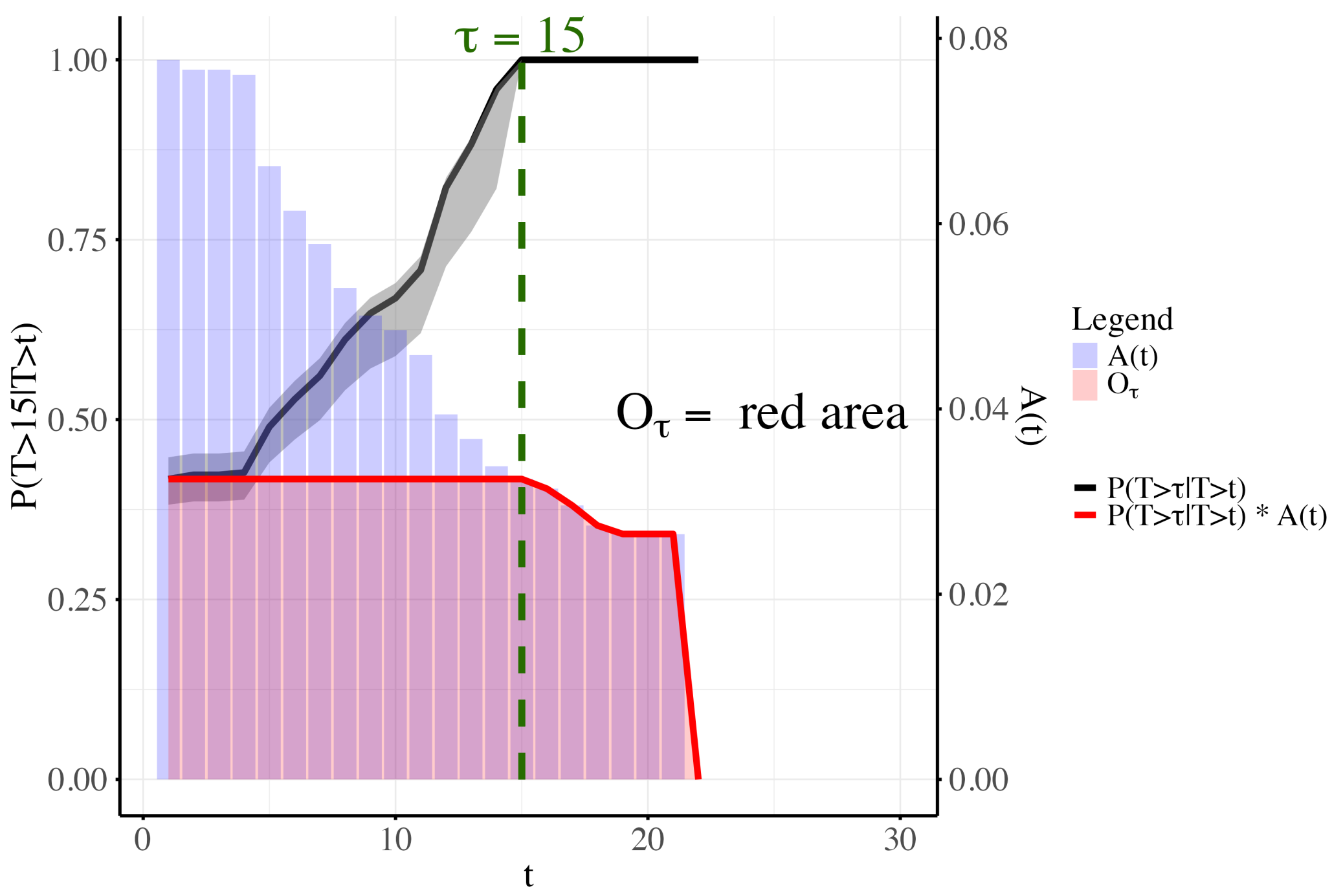

Fig. S1: Estimation of O_τ for the arbitrary case of $\tau = 15$ (green dashed line). It shows the conditional survival $P(T > \tau \mid T > t)$ (black line, 95% CI in grey), the age distribution $A(t)$ (blue histogram), the t-wise evaluation of $P(T > \tau \mid T > t) \cdot A(t)$ (red line), and the value of $O_\tau = \sum_{t=1}^{\infty} P(T > \tau \mid T > t) \cdot A(t)$ (red area; in practice the sum is over $t = 1, \ldots, t_{\max}$).

Computing O_τ requires a choice of τ to be made (in Figure S1, the arbitrary choice was $\tau = 15$). Alternatively, O_τ can be plotted as a function of τ, producing a curve that we have called $O(\tau)$. This curve—as opposed to the survival function $S(t)$, which illustrates the probability *at birth* of surviving beyond age t—illustrates the probability *at the age of sampling* of surviving beyond age τ, where the age at sampling is estimated as an $A(t)$-weighted average of all sampleable ages.

Estimating $A(t)$

In order to simulate the process of randomly sampling an individual from a population, an estimation of its age distribution $A(t)$ is required. The age distribution of a wild population can be challenging to measure directly, and therefore an estimation may be necessary. One way of achieving this is from survival and fertility data. If the population is stable (i.e. constant age-specific birth and death rates, and no migration), $A(t)$ can be shown to converge to a stable equilibrium (**?**). This time-invariant $A(t)$ can be calculated by constructing the Leslie matrix (which summarises age-specific fertility and mortality) and projecting it forward in time (**?**), or by calculating the eigenvector of its dominant eigenvalue (**?**). A special case of stable population is a stationary one (i.e. no growth and no migration), which has $A(t)$ equal to its normalised survival function $S(t)$ (**?**).

Wild populations are rarely stable, however, due to density-dependent and stochastic environmental processes. These can be modelled by specifying Leslie matrices that are themselves functions of time or of population size (**?**). The age distribution of a non-stable

population is not guaranteed to converge to a stable equilibrium: convergence to periodic or chaotic attractors is also possible (**??**).

Since the objective of estimating $A(t)$ is to simulate the process of randomly sampling an individual of age t, an alternative is to simulate this process directly from empirical data. If the ages of a random sample of individuals from the population can be determined, then $A(t)$ can be approximated as the normalised distribution of the age frequencies of this sample. Additionally, methods have also been proposed to estimate $A(t)$ from capture-recapture data of wild individuals when the age at first capture is unknown (**??**).

Propagation of uncertainty

The uncertainty around the metric O_τ is propagated from the uncertainty of the KM estimator of $S(t)$. In this section, this propagation is explicitly demonstrated.

Uncertainty around conditional survival

The KM estimator is fitted in R using the `survival::survfit` function, which allows for the argument `conf.int` to be set to the desired confidence level (default is 0.95).

The TAUS method presents a 95% confidence interval for the conditional probability of an individual of age t outliving age τ, $P(T > \tau \mid T > t)$ (see black line in Figure S1). This conditional survival is calculated as a ratio of bounded quantities, as described in Equation S5, where $S(t)$ is estimated using a standard KM model. For a ratio $R = \frac{A}{B}$ with $A, B > 0$; R is largest when A is at its upper bound and B at its lower bound, and smallest when A is at its lower bound and B at its upper bound. Thus, for a ratio $R = \frac{A}{B}$, the bounds of R are given by:

$$U_R = \frac{U_A}{L_B} \tag{S7}$$

$$L_R = \frac{L_A}{U_B} \tag{S8}$$

Here $P(U_R)$ denotes the probability that the upper bound of R is attained (i.e. A at its upper bound and B at its lower bound), and similarly $P(L_R)$ denotes the probability that the lower bound of R is attained. If the original bounds represent the X% CI around the original quantities A and B, and the two tail events are treated as independent, then $P(U_R) = P(U_A \cap L_B) = P(U_A)\,P(L_B) = (\frac{1-X}{2})^2$, and similarly for $P(L_R)$. Thus the ratio bounds correspond to a $(\frac{1-X}{2})^2$ probability in each tail:

$$P(U_R) = P(U_A \cap L_B) = \left(\frac{1-X}{2}\right)^2 \tag{S9}$$

$$P(L_R) = P(L_A \cap U_B) = \left(\frac{1-X}{2}\right)^2 \tag{S10}$$

Hence, when initially computing the KM estimator, the confidence intervals must be chosen such that their ratio will yield the desired 95% CI for the conditional survival. For a 95% CI, each tail has probability 0.025; we therefore set $\left(\frac{1-X}{2}\right)^2 = 0.025$ and solve for X:

$$P(U) = P(L) = \frac{1 - 0.95}{2} = 0.025 \tag{S11}$$

$$\left(\frac{1 - X}{2}\right)^2 = 0.025 \tag{S12}$$

$$X = 1 - 2\sqrt{0.025} \tag{S13}$$

In summary, by setting `conf.int = 1-2*sqrt(0.025)` in the call to `survival::survfit`, the conditional survival bounds computed as seen in Equation S7 and Equation S8 define a 95% CI for $P(T > \tau \mid T > t)$.

Uncertainty around O_τ

The probability of a random individual of unknown age outliving age τ (i.e. O_τ) is also presented with a 95% CI. O_τ is calculated as the weighted sum of the conditional survival for all possible values of t (see Equation S6).

The uncertainty around this metric is propagated from the uncertainty of the conditional survival alone, since $A(t)$ (the age distribution) is treated like a fixed probability distribution. Because O_τ is a weighted sum (Equation S6) and each term is positive, the upper (lower) bound of the sum is the sum of the upper (lower) bounds of each term. Thus, the bounds of O_τ are given by:

$$U_{O_\tau} = \sum_{t=1}^{t_{\max}} U_{P(T>\tau|T>t)} \cdot A(t) \tag{S14}$$

$$L_{O_\tau} = \sum_{t=1}^{t_{\max}} L_{P(T>\tau|T>t)} \cdot A(t) \tag{S15}$$

Statistical analysis

Once O_τ has been computed for two populations, it is often of ecological relevance to test for a statistically significant difference. Here, we propose a pairwise test for two O_τ values and their 95%CI intervals that uses p-values to express statistical difference.

Since O_τ is a probability, we chose to model the pairs as Beta-distributed random variables, and calculated the distance between their distributions using the Kolmogorov-Smirnov (KS) test. Then, a p-value was computed through bootstrapping to assess the statistical significance of the KS distance between the two distributions. This section explains the implementation of this test in detail.

The Beta distribution is a continuous probability distribution defined in the interval $[0, 1]$, and it is commonly used to model probabilities. It has two shape parameters, α and β. The mean and variance of a Beta-distributed random variable $X \sim \text{Beta}(\alpha, \beta)$ are given by:

$$X \sim \text{Beta}(\alpha, \beta) \Rightarrow \begin{cases} \mathbb{E}[X] = \frac{\alpha}{\alpha+\beta} \\ \text{Var}(X) = \frac{\alpha\beta}{(\alpha+\beta)^2(\alpha+\beta+1)} \end{cases} \tag{S16}$$

Following this definition, O_τ can be modelled by setting $\mathbb{E}(X) \approx O_\tau$ and $\mathrm{Var}(X) \approx (U-L)^2$, where U and L are the upper and lower limits of the 95% CI for O_τ, respectively (see Section S1.4 for the calculation of these). Writing $\mu = \mathbb{E}[X]$ and $\sigma^2 = \mathrm{Var}(X)$, Equation S16 gives $\mu = \frac{\alpha}{(\alpha+\beta)}$ and $\sigma^2 = \frac{\alpha\beta}{((\alpha+\beta)^2(\alpha+\beta+1))}$. Solving for α and β in terms of μ and σ^2 yields $\alpha = \mu(\frac{\mu(1-\mu)}{\sigma^2} - 1)$ and $\beta = (1-\mu)(\frac{\mu(1-\mu)}{\sigma^2} - 1)$. Substituting $\mu = O_\tau$ and $\sigma^2 = (U-L)^2$ gives:

$$\alpha = O_\tau^2\left(\frac{1-O_\tau}{(U-L)^2} - \frac{1}{O_\tau}\right), \qquad \beta = \alpha\left(\frac{1}{O_\tau} - 1\right) \tag{S17}$$

Once the Beta distributions are defined, the KS distance between them is the maximum vertical difference between their two cumulative functions:

$$D = \sup_x |F_1(x) - F_2(x)| \tag{S18}$$

where $F_1(x)$ and $F_2(x)$ are the cumulative distribution functions of the two Beta distributions being compared, as evaluated at x.

In order to test the statistical significance of this distance D, we first fit a null Beta distribution to both values of O_τ under comparison. We estimate the null distribution by averaging the α and β parameters of the two independently-fitted Beta distributions:

$$\mathrm{Beta}_0 = \mathrm{Beta}(\frac{\alpha_1 + \alpha_2}{2}, \frac{\beta_1 + \beta_2}{2}) \tag{S19}$$

where α_1, β_1, α_2 and β_2 are the parameters of the fitted Beta distributions of the two O_τ values under comparison. We then draw pairs of samples from Beta_0 and calculate their KS distance using the R function `stats::ks.test`. We repeat this process B times and store the results (i.e. bootstrap). A p-value is then computed as the proportion of these KS distances that are larger than the numerically computed D (Equation S18).

This number is the probability of obtaining a KS distance D or larger under the null hypothesis that the two O_τ values come from the same Beta distribution (i.e. they are not statistically different).

S2. EXAMPLE 2 - FLIES AT DIFFERENT TEMPERATURES

The dataset used in this section (which is publicly available (**?**)) contains data on fly survival, strain, and rearing temperature. Similar to the previous example, some variables were removed to keep the case study focused on the analysis of how a single variable affected survival. After pre-processing, the dataset contained survival data on *Drosophila* flies reared at different temperatures. Graphical representation of the observed events (deaths and censoring, Figure S2a), and the corresponding KM curves (Figure S2b), show that higher temperatures lead to higher mortality at younger ages, although individuals with extremely long lifespans ($>$ 200 days) were present in all groups.

Cox PH

A Cox model was fitted to these data with temperature as the only covariate for survival times. The model was found, again, to be invalid due to the violation of the PH assumption

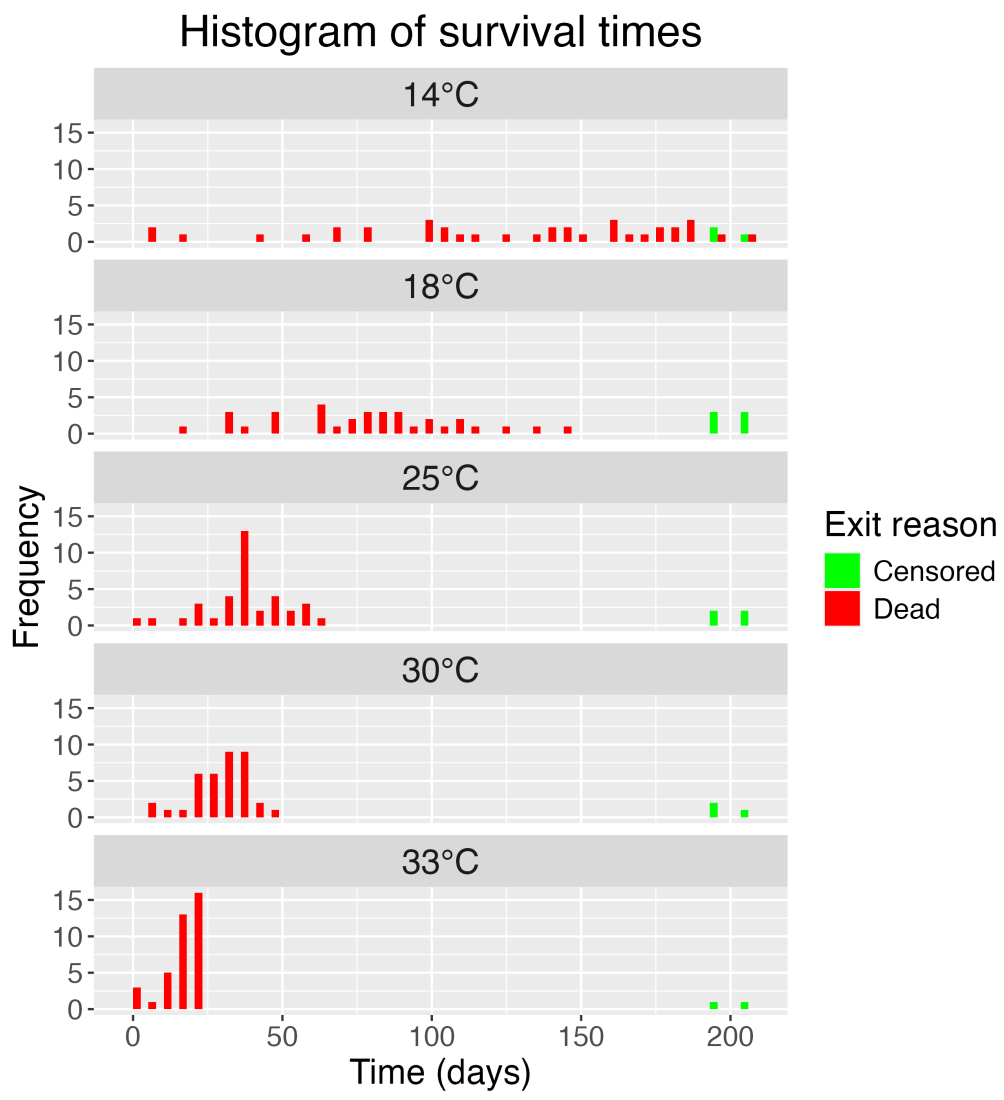

(a) Histogram of times recorded

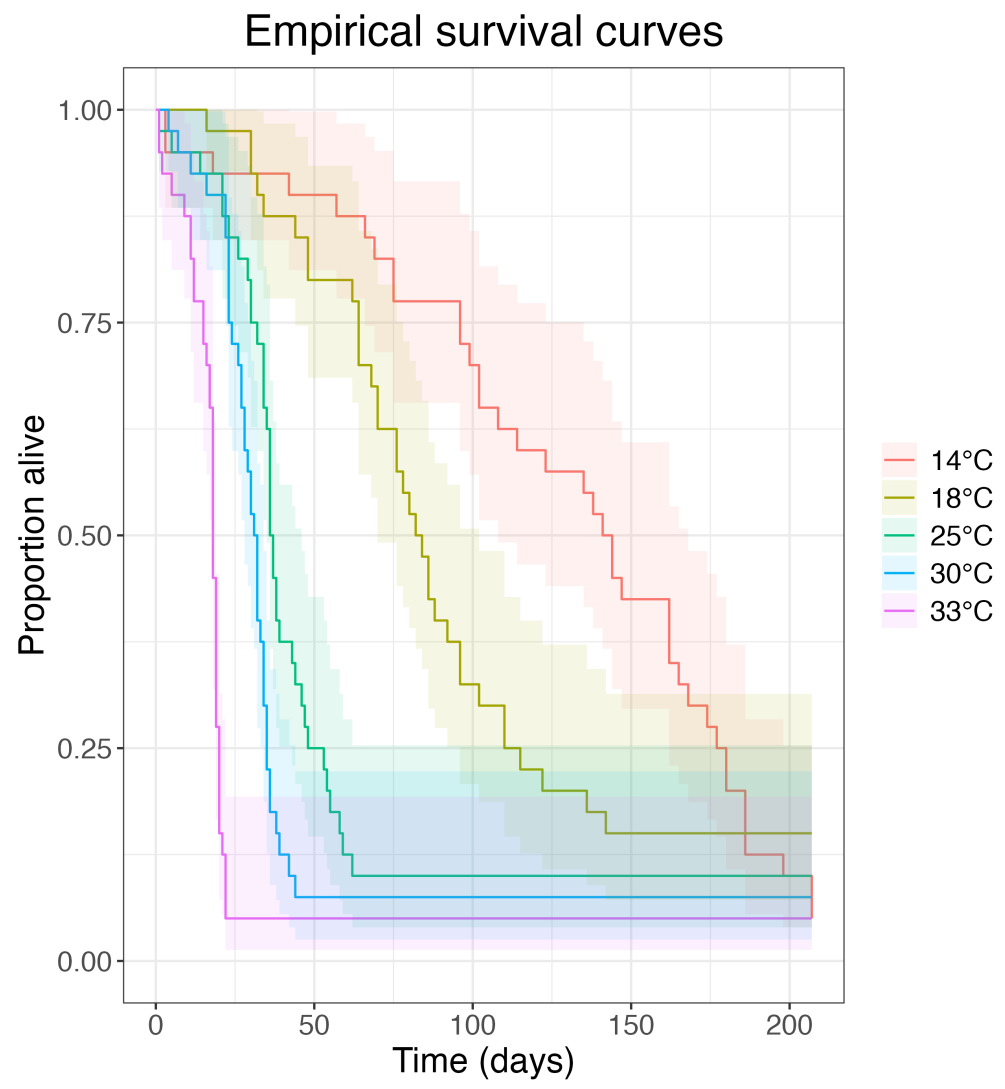

(b) KM curves

Fig. S2: Example 2 - data description. (a) Distribution of event times of the five populations in the study. The x-axis represents the age of the individuals, while the y-axis represents the number of individuals at each age. Red bars represent individuals that were followed up until death and green bars represent individuals whose follow-up ended for a different reason (censored), in this case due to the end of the experiment. (b) Kaplan-Meier curves for each population in the study. The x-axis represents time, and the y-axis represents survival probability at birth.

($p << 0.05$), and thus its output was not further investigated. However, and for completion and comparison purposes, it is reported in Table S1.

Parametric survival

The most appropriate distribution for the parametric modelling of these survival data was the Log-logistic, which captured the most obvious general trend in the effect of temperature on *Drosophila* survival — that is, that higher temperatures increase early-life mortality (Figure S3). However, the model fails to fully capture the early-life survival dynamics, and suggests an artificial temperature-driven change in the proportion of extremely long-lived individuals. Additionally, it offers arguably poor estimates of the slopes of some of the empirical survival curves (Figure S3).

Table S1: Output of the Cox PH model applied to the dataset from Example 2: Flies at different temperatures.

**Variable**	**HR** $(\exp(\beta))$	**95% CI**	**p-value**
Temperature (18°C)	1.2	0.73 – 1.9	0.50
Temperature (25°C)	2.2	1.4 – 3.6	8.3×10^{-4}
Temperature (30°C)	3.2	2.0 – 5.2	1.1×10^{-6}
Temperature (33°C)	7.9	4.9 – 13.0	$< 2.0 \times 10^{-16}$
Reference level: Temperature = 14°C			
Model statistics: $n = 200$, events = 182			
Concordance = 0.772 (SE = 0.02)			
Likelihood ratio test = 78.9 on 4 df, $p = 3.0 \times 10^{16}$			
Wald test = 89.5 on 2 df, $p < 2.0 \times 10^{16}$			
Score (logrank) test = 104.9 on 2 df, $p < 2.0 \times 10^{16}$			

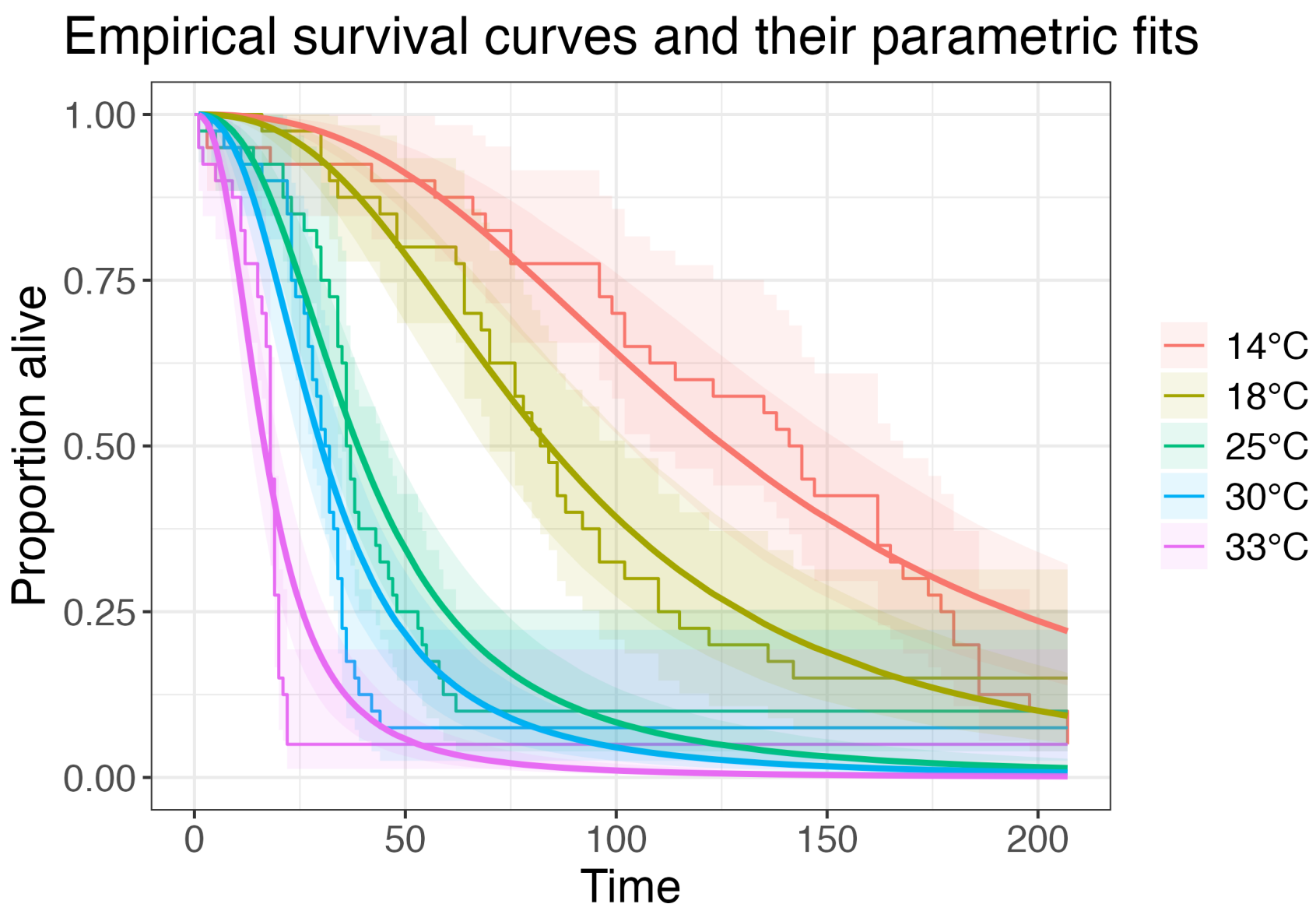

Fig. S3: KM curves of each population in the study (step curves), overlapped with their respective parametric fits (smooth curves).

Fitting a Log-logistic model to survival data computes an Accelerated Failure Time (AFT) model, which models survival effects of covariates as dilations or contractions of the (log-) time dimension (**?**). In the case of the Log-logistic model, this is achieved by estimating the change in the scale parameter (α, the median of the distribution, or the age at which 50% of the population has died) as a linear function of the explanatory variables. Thus, fitting a Log-logistic parametric model to these data gives us information on how much faster individuals experience the passage of time under different rearing temperatures (Table S2, Figure S3). However, the effect of rearing temperature on *Drosophila* survival seems more complex than a simple time dilation/contraction: although the majority of the population appears to experience such effect, the proportion of extremely long-lived individuals seems to be irresponsive to their rearing temperature (Figure S2b).

This could cause misleading conclusions in certain ecological contexts. For example, in the context of R-selected species such as *Drosophila*, individuals with the longest lifespans are arguably the most relevant for population persistence in the wild, since they will undergo the most gonotrophic cycles and thus contribute the most to population growth. In an epidemiological context, extremely long-lived individuals are also likely to become superspreaders and thus contribute disproportionately to disease transmission. In both cases, a survival model that fails to capture the effects of the covariates of interest on the proportion of extremely long-lived individuals can lead to conclusions that deviate from reality in ecologically meaningful ways. This is the case of this Log-logistic parametric model, which wrongly predicts that warmer temperatures will result in a lower proportion of individuals living extremely long lives, despite this not being supported by the empirical survival curves (Figure S3).

Table S2: Output of parametric survival model fitted to the data in Example 2. The table shows the effect on the Log-logistic's distribution location parameter (the scale parameter) of each temperature group against the reference group (14°C). This table is a modified version of the output that can be obtained calling the R function `flexsurv::tidy()` on the fitted model object.

Variable	Estimate(β)	exp(β)	Interpretation	p-val
shape	2.53	NA	NA	NA
scale	126.0	NA	NA	NA
temp18°C	-0.401	0.669	Survival time is 67% of baseline	0.006
temp25°C	-1.18	0.307	Survival time is 31% of baseline	<0.001
temp30°C	-1.43	0.240	Survival time is 24% of baseline	<0.001
temp33°C	-2.03	0.131	Survival time is 13% of baseline	<0.001

TAUS

These empirical data differ from the previous example in that, for most groups, the last death recorded was a long time before the end of the follow-up period (Figure S2a). This can introduce strong survivorship bias: when most of the population dies at early ages, the longest-lived individuals account for a large proportion of the population, increasing their odds of being (randomly) sampled. This leads to the counterintuitive situation in which the probability of a randomly-sampled individual outliving old ages is highest when early mortality is also highest. The extent of the survivorship bias on this dataset can be appreciated in the conditional survival matrices (Figure S4).

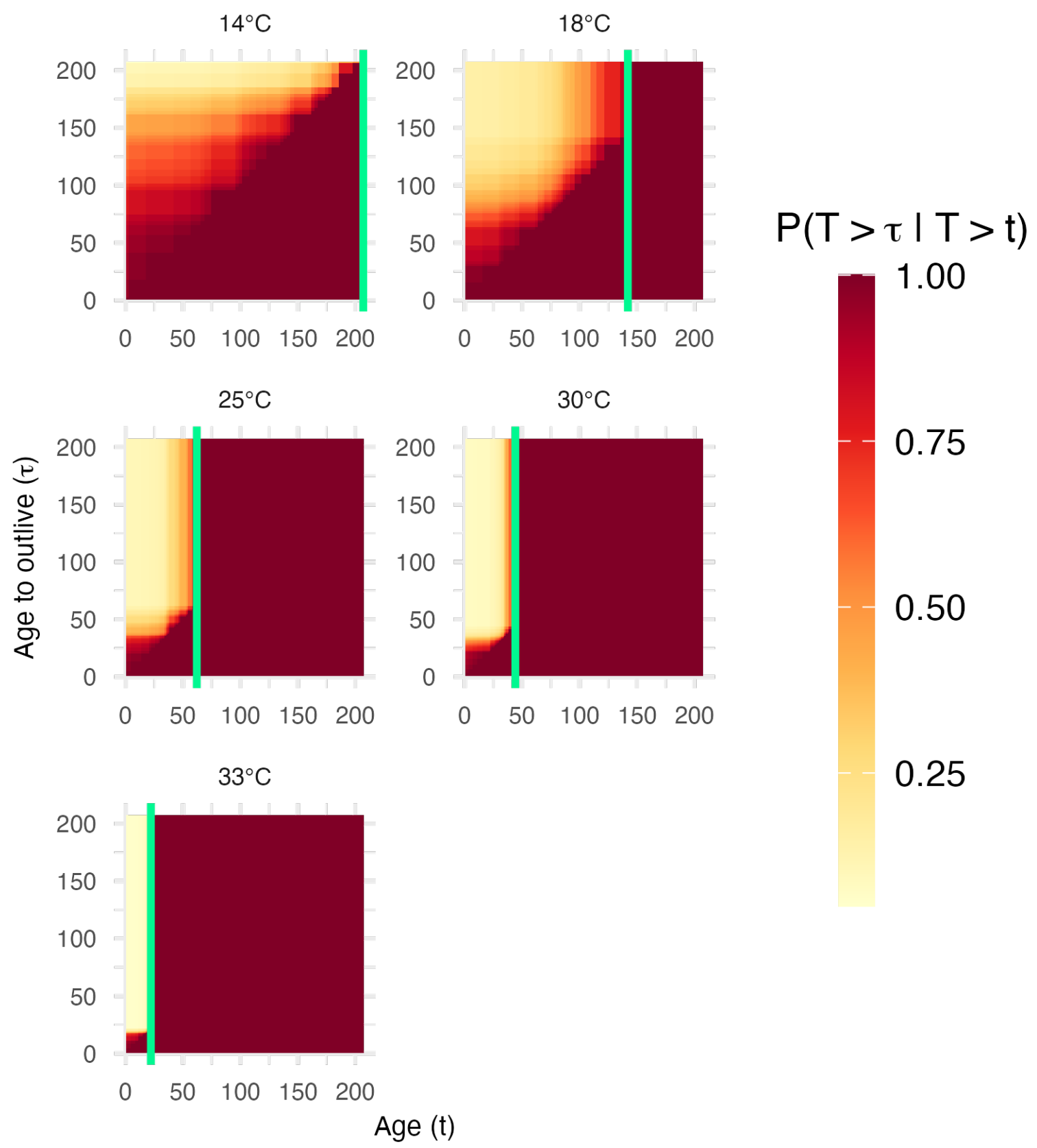

Fig. S4: Each panel shows the conditional survival matrix for one of the groups. The x-axes represent the current age of the individuals (t), while the y-axes represent the age beyond which survival is of interest (τ). The colour of each cell (x, y) represents the probability that an individual of age $t = x$ will outlive age $\tau = y$, that is, $P(T > \tau \mid T > t)$. Grey cells represent coordinates (t, τ) for which this probability is undefined (i.e. $\tau, t \geq$ maximum age observed in the group). The vertical green lines mark the time of the last death observed in each group.

The consideration of the effects of the survivorship bias by the TAUS method is propagated from the conditional survival matrices. The $O(\tau)$ curves (i.e. the probability of a randomly sampled individual outliving age τ as a function of τ) show this very clearly (Figure S5a). Let us consider the extreme case of the 33°C group: most individuals died before age $\tau \approx 25$, but the remaining minority outlived the duration of the experiment, all of them reaching age $\tau > 200$ (Figure S2a). This means that the probability of randomly sampling one of these extremely long-lived individuals is disproportionately high. As a result, individuals from this group have the highest $O(\tau)$ values for high values of τ (Figure S5a).

Thanks to the integration of the survivorship bias, TAUS is able to capture the general survival dynamics of the system: rearing temperatures impact early life survival (e.g. $\tau = 43$, Figure S5b, Table S3), but do not affect the proportion of extremely long-lived individuals (e.g. $\tau = 125$, Figure S5c, Table S3); as suggested by the histogram of event times (Figure S2a). In contrast, Cox and parametric models did not capture this (Tables S1, S2; Figure S3).

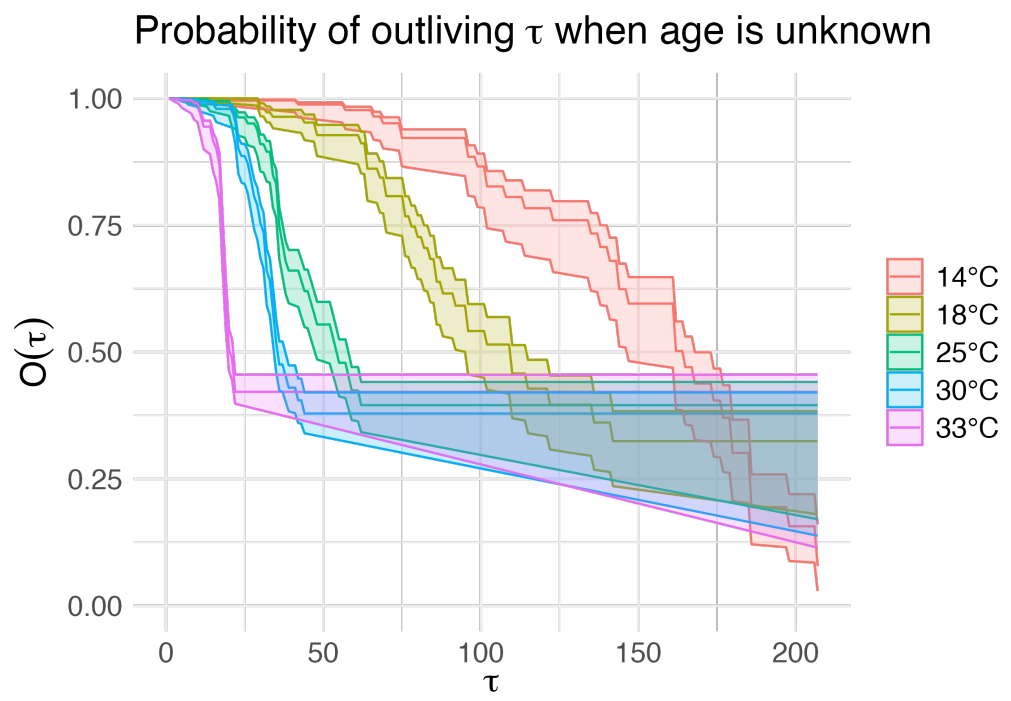

(a) Probability of outliving τ when the age is unknown, as a function of τ, $O(\tau)$

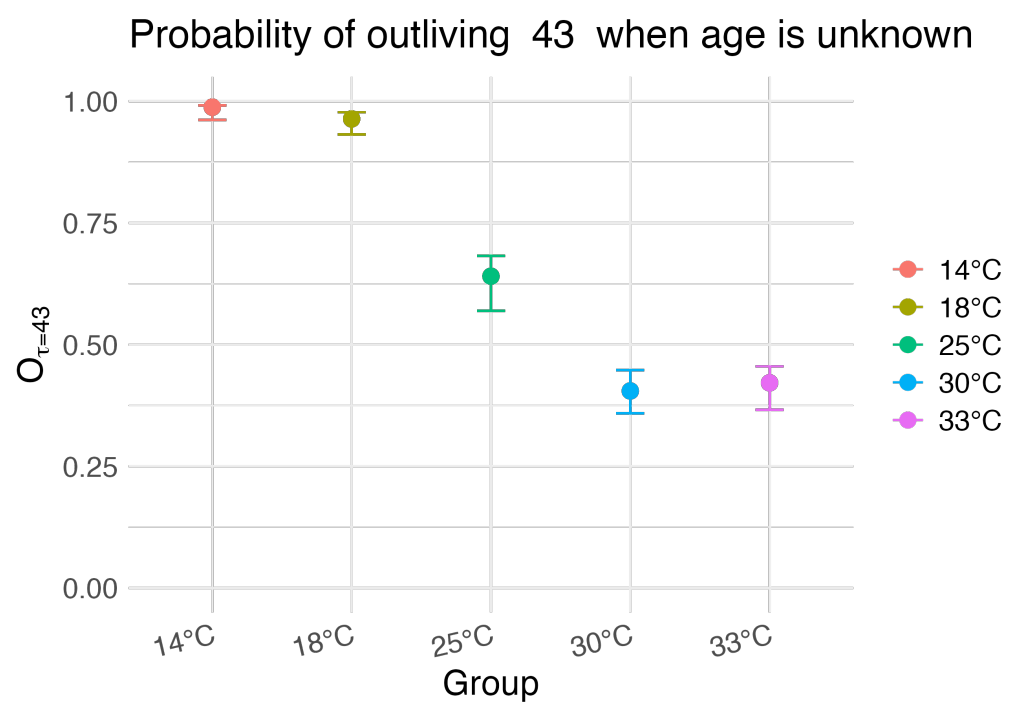

(b) Probability of outliving $\tau = 43$ when the age is unknown, $O(43)$

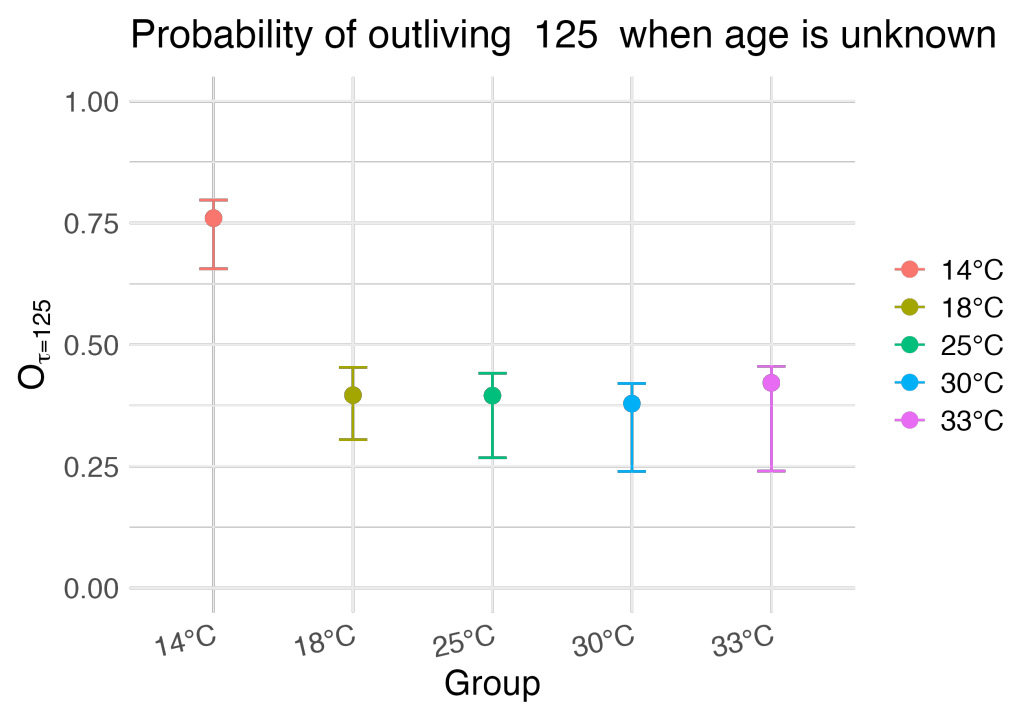

(c) Probability of outliving $\tau = 125$ when the age is unknown, $O(125)$

Fig. S5: Outputs of the TAUS method describing the survival dynamics of flies reared at different temperatures. (a) The probability of outliving age τ as a function of τ of a randomly sampled individual of unknown age. (b) The probability of outliving age $\tau = 43$ of a randomly sampled individual of unknown age. (c) The probability of outliving age $\tau = 125$ of a randomly sampled individual of unknown age. Shaded region (a) and error bars (b and c) show the 95% CI bounds.

Table S3: Pairwise comparisons of $O(\tau)$

Comparison		$O(\tau = 43)$		$O(\tau = 125)$	
**Group 1**	**Group 2**	**p-val**	**Reject H_0?**	**p-val**	**Reject H_0?**
14°C	18°C	0.167	No	0.002	Yes
14°C	25°C	<0.001	Yes	0.001	Yes
14°C	30°C	<0.001	Yes	0.003	Yes
14°C	33°C	<0.001	Yes	0.010	Yes
18°C	25°C	<0.001	Yes	1.000	No
18°C	30°C	<0.001	Yes	1.000	No
18°C	33°C	<0.001	Yes	0.993	No
25°C	30°C	0.002	Yes	1.000	No
25°C	33°C	0.002	Yes	1.000	No
30°C	33°C	1.000	No	0.994	No

CODE AVAILABILITY

The code used to simulate the data and to analyse the datasets is available in the supplementary materials and as the R package `TAUS` at https://github.com/casasgomezuribarri/TAUS (branch `package`).

REFERENCES

Caswell, H. (2001). *Matrix Population Models: Construction, Analysis and Interpretation*. Sinauer Associates, Sunderland, MA, 2nd edition.

Chu, C. C. (2015). Population dynamics: Theory of nonstable populations. *International Encyclopedia of the Social & Behavioral Sciences: Second Edition*, pages 581–583.

Gerken, A. (2023). Data from: Demographic parameters shift over time among artificially selected drosophila melanogaster: a case for or against lifetime reproductive measurements - catalog.

Howard, R. (2026). A note on the eigenvalues and eigenvectors of leslie matrices. Lecture notes, Department of Mathematics, University of South Carolina.

Inaba, H. (2017). *Age-Structured Population Dynamics in Demography and Epidemiology*. Springer Nature.

Jackson, C., Metcalfe, P., Amdahl, J., Warkentin, M. T., Sweeting, M., and Kunzmann, K. (2011). flexsurv: Flexible parametric survival and multi-state models. R package.

Lotka, A. J. (1937). Population analysis: a theorem regarding the stable age distribution. *Journal of the Washington Academy of Sciences*, 27:299–303.

Müller, H. G., Wang, J. L., Carey, J. R., Caswell-Chen, E. P., Chen, C., Papadopoulos, N., and Yao, F. (2004). Demographic window to aging in the wild: Constructing life tables and estimating survival functions from marked individuals of unknown age. *Aging Cell*, 3:125–131.

Müller, H. G., Wang, J. L., Yu, W., Delaigle, A., and Carey, J. R. (2007). Survival and aging in the wild via residual demography. *Theoretical Population Biology*, 72:513–522.
